# Nasal Colonisation by *Staphylococcus aureus* Depends upon Clumping Factor B Binding to the Squamous Epithelial Cell Envelope Protein Loricrin

**DOI:** 10.1371/journal.ppat.1003092

**Published:** 2012-12-27

**Authors:** Michelle E. Mulcahy, Joan A. Geoghegan, Ian R. Monk, Kate M. O'Keeffe, Evelyn J. Walsh, Timothy J. Foster, Rachel M. McLoughlin

**Affiliations:** 1 Department of Microbiology, Moyne Institute of Preventive Medicine, Trinity College Dublin, Dublin, Ireland; 2 School of Biochemistry and Immunology, Trinity Biomedical Sciences Institute, Trinity College Dublin, Dublin, Ireland; University of Tubingen, Germany

## Abstract

*Staphylococcus aureus* asymptomatically colonises the anterior nares, but the host and bacterial factors that facilitate colonisation remain incompletely understood. The *S. aureus* surface protein ClfB has been shown to mediate adherence to squamous epithelial cells *in vitro* and to promote nasal colonisation in both mice and humans. Here, we demonstrate that the squamous epithelial cell envelope protein loricrin represents the major target ligand for ClfB during *S. aureus* nasal colonisation. *In vitro* adherence assays indicated that bacteria expressing ClfB bound loricrin most likely by the “dock, lock and latch” mechanism. Using surface plasmon resonance we showed that ClfB bound cytokeratin 10 (K10), a structural protein of squamous epithelial cells, and loricrin with similar affinities that were in the low µM range. Loricrin is composed of three separate regions comprising GS-rich omega loops. Each loop was expressed separately and found to bind ClfB, However region 2 bound with highest affinity. To investigate if the specific interaction between ClfB and loricrin was sufficient to facilitate *S. aureus* nasal colonisation, we compared the ability of ClfB^+^ *S. aureus* to colonise the nares of wild-type and loricrin-deficient (Lor^−/−^) mice. In the absence of loricrin, *S. aureus* nasal colonisation was significantly impaired. Furthermore a ClfB^−^ mutant colonised wild-type mice less efficiently than the parental ClfB^+^ strain whereas a similar lower level of colonisation was observed with both the parental strain and the ClfB^−^ mutant in the Lor^−/−^ mice. The ability of ClfB to support nasal colonisation by binding loricrin *in vivo* was confirmed by the ability of *Lactococcus lactis* expressing ClfB to be retained in the nares of WT mice but not in the Lor^−/−^ mice. By combining *in vitro* biochemical analysis with animal model studies we have identified the squamous epithelial cell envelope protein loricrin as the target ligand for ClfB during nasal colonisation by *S. aureus*.

## Author Summary

*Staphylococcus aureus* is an important human commensal, present permanently in the noses of about 20% of the population and representing a significant risk factor for infection. The host and bacterial factors that facilitate nasal colonisation remain to be fully characterised. *S. aureus* adheres to the squamous epithelial cells found in the nose. Proteins expressed on the surface of *S. aureus*, including clumping factor B (ClfB), are responsible for this interaction. We demonstrate that loricrin, a major component of the squamous epithelial cell envelope, represents the primary ligand for ClfB and that the interaction between ClfB and loricrin is required for efficient nasal colonisation by *S. aureus*. Using purified proteins we have demonstrated that ClfB binds loricrin and propose a mechanism by which this binding occurs. We have established a murine model of *S. aureus* nasal colonisation and have demonstrated reduced colonisation in loricrin-deficient mice compared to wild-type mice which is dependent upon ClfB. Using *Lactococcus lactis* as a surrogate host expressing ClfB, we could show that the interaction between ClfB and loricrin in the nares is sufficient to support nasal colonisation. Cumulatively, these data show that the ClfB-loricrin interaction is crucial for nasal colonisation by *S. aureus*.

## Introduction

*Staphylococcus aureus* is a commensal of humans that permanently colonises the anterior nares of about 20% of the population with the remainder being colonised transiently [1]. The bacterium is an opportunistic pathogen that can cause a variety of disorders ranging in severity from superficial skin lesions to more serious invasive and life-threatening infections such as endocarditis and septicaemia. Nasal carriage is an established risk factor for *S. aureus* infections both in the hospital and in the community with individuals often being infected with the strain that they carry [2]–[4]. Nasal carriage can be transiently eradicated by topical administration of the antibiotic mupirocin but this is compromised by the development of resistance [5]. Alternative strategies for reducing nasal carriage are required which will involve a detailed understanding of the molecular basis of interactions between the host and the bacterium that underlie the process.

Host factors that determine nasal colonisation are incompletely understood. Polymorphisms in the genes encoding the glucocorticoid receptor, C-reactive proteins, interleukin-4 and complement inhibitor proteins have been associated with persistent nasal carriage [6]–[8]. In addition, reduced expression of antimicrobial peptides in nasal secretions is associated with nasal carriage [9]. The normal flora can also influence the ability of *S. aureus* to colonise the nares [10]–[11].

A fundamental feature that likely dictates the interaction between *S. aureus* and the host during nasal colonisation is adhesion of bacteria to nasal epithelial surfaces, a process which depends upon specific interactions between adhesins on the bacterial cell surface and their target ligands in the epithelium. The primary habitat of *S. aureus* in colonised individuals is the moist squamous epithelium in the anterior nares [12]–[13]. The outer part of this epithelial surface, known as the *stratum corneum*, consists of layers of dead keratinized cells called squames. Keratinocytes in the basal layer are continuously dividing. As cells migrate toward the *stratum corneum* they differentiate into squames, a process which involves expression of proteins that will eventually form the cornified envelope (CE) that replaces the cytoplasmic membrane in these cells. The CE is composed of proteins such as loricrin, involucrin and small proline-rich proteins that are extensively cross-linked, in addition to ceramides that are attached both covalently and non-covalently [14]–[16]. The extensive cross-linking in addition to conformational properties makes the CE a highly resilient structure that plays an important role in barrier function [17]. Loricrin is the most abundant protein of the CE forming about 80% of the protein mass [18]. Cytokeratins 1 and 10 are present on the interior of squames and are exposed on their surface [19]. Despite the importance of the CE in barrier function and the fact that loricrin is highly abundant in the CE of squames, it is surprisingly non-essential. A loricrin knock-out mouse has been generated and while these mice demonstrate a delay in the formation of the skin barrier in embryonic development, by days 4–5 after birth the skin phenotype disappears. Loricrin-deficient (Lor^−/−^) mice breed normally and appear phenotypically indistinct from wild-type litter mates [20]. The absence of a more severe phenotype in these mice is due to the existence of a compensatory loricrin back-up system. Increased expression of small proline rich proteins has been observed in the CE of Lor^−/−^ mice. Interestingly expression of other CE components such as involucrin, filaggrin and Cytokeratin 10 (K10) were similar in Lor^−/−^ and wild-type mice [21].

Two *S. aureus* surface proteins, clumping factor B (ClfB) and iron regulated surface determinant A (IsdA), have been strongly implicated in nasal colonisation. By comparing wild-type strains with isogenic mutants lacking the proteins, both ClfB and IsdA were shown to promote adhesion to squames *in vitro* [19], [22] in addition to promoting colonisation of the nares of rodents [22]–[23] and in the case of ClfB, humans [24]. *S. aureus* surface protein G (SasG) and the serine-aspartate repeat proteins SdrC and SdrD also promote bacterial adhesion to squames *in vitro* [25] but their roles, if any, *in vivo* have not yet been tested.

ClfB is a member of a family of proteins that are structurally related to clumping factor A (ClfA), the archetypal fibrinogen (Fg) binding protein of *S. aureus*. It is attached covalently to peptidoglycan in the cell wall by sortase. The C-terminal cell wall anchorage domain comprises an LPXTG sortase cleavage motif, a hydrophobic membrane-spanning region followed by a stretch of positively charged residues (Figure S1A) [26]. The N-terminal 542 residues comprise the ligand-binding A domain followed by a flexible stalk formed by repeats of the dipeptide serine-aspartate. The A domain is composed of three separately folded subdomains N1, N2 and N3, the last two of which are the minimum required for binding to ligands Fg and K10 [27]–[29].

The binding site for ClfB in Fg is a single repeat (number 5) in the αC region of the α-chain [30]. In addition the protein binds to the C-terminus of K10 which is composed of quasi repeats of the amino acid sequence Y[GS]_n_Y [19]. This type of sequence can form omega loops where the Y residues bind to each other by hydrophobic interactions resulting in the GS sequences protruding as loops forming rosette-like structures [31]–[32]. One such omega loop sequence (YGGGSSGGGSSSGGGY) was shown to bind to recombinant ClfB A domain with a K*_D_* in the low micromolar range [33]. Recently the X-ray crystal structures of both the apo form of ClfB N2N3 and the protein in complex with peptides mimicking the binding domains in Fg and K10 were solved [28]–[29]. These studies demonstrated that the two seemingly disparate proteins contain related peptides that can bind in a hydrophobic trench located between the separately folded N2 and N3 domains. In each study, similar consensus motifs (GSSGXG, [28], GSSGG/S/TGXXG, [29]) were defined for Fg and K10 with residues in the trench forming bonds with each of the peptide residues [28]. These studies confirmed earlier predictions that ClfB bound its ligands by the “dock, lock and latch” mechanism first defined for the Fg binding proteins SdrG and ClfA [34]–[35]. After the peptide inserts into the ligand-binding trench a C-terminal extension of domain N3 undergoes a conformational change, covers the inserted peptide and binds residues in N2 by β-strand complementation which locks the peptide in place.

Loricrin is the major structural protein of the CE of squames [16], [36]–[37]. Human loricrin comprises sequences capable of forming GS-rich omega loops similar to those in K10. Located between the loop domains and also at the N- and C-termini are stretches rich in glutamate and cysteine residues that form covalent links to other proteins in the CE by transglutamination and disulfide bond formation [36], [38]. Polymorphisms can occur in the loricrin gene in humans that result in loss of four residues within loop region 2 [39]. The GS-rich omega loop composition of loricrin and its abundance in the CE of squames suggested to us that it could serve as an important ligand for ClfB. Previous studies have identified K10 as a ligand for ClfB *in vitro*. It has been assumed that K10 is the element on squames to which ClfB binds and that this interaction is an important determinant of nasal colonisation. However, this remains only an association and has not been proven unambiguously.

Herein we identify a novel interaction between *S. aureus* ClfB and loricrin that is critically required for *S. aureus* nasal colonisation. *S. aureus* was shown to adhere to immobilized human and murine loricrin in a ClfB-dependent fashion. The affinity of recombinant ClfB for human and murine loricrin was comparable to K10 and Fg by surface plasmon resonance. Wild-type and Lor^−/−^ mice were inoculated intra-nasally with ClfB-expressing bacteria to investigate the role of loricrin *in vivo* and we demonstrate that a specific interaction between ClfB and loricrin occurs during the colonisation process. We conclude that loricrin is the major ligand for ClfB in the nares of mice.

## Results

### ClfB promotes adherence of *S. aureus* to loricrin

ClfB is known to bind to the C-terminal “tail” region of human and murine K10 which is composed of quasi repeats of Y[GS]_n_Y, sequences which can form omega loops [31], [33]. Loricrin, the major component of the CE of squames, is almost entirely composed of similar structures. In order to investigate if ClfB binds loricrin and to dissect the binding domains within the protein, DNA encoding human and murine loricrin was synthesized and cloned into the expression vector pGEX, so that N-terminally GST-tagged proteins could be expressed and purified.

**Figure 1:**
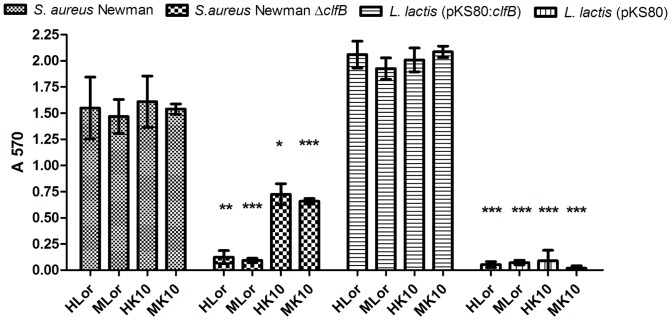
ClfB promotes adherence of *S. aureus* Newman and *L. lactis* to loricrin. *S. aureus* Newman, Newman Δ*clfB*, *L. lactis* MG1363 (pKS80) and *L. lactis* MG1363 (pKS80:*clfB*) were tested for binding to ligands immobilized on 96-well plates. Bacteria were added to wells coated with immobilized GST-tagged recombinant human loricrin (Hlor), murine loricrin (MLor)) human K10 peptide (HK10) and murine K10 peptide (MK10)) (1 µM). Bacterial adherence was measured by staining with crystal violet and measurement of the absorbance at 570 nm. Values represent the mean ± SD of triplicate wells. The data shown is representative of two individual experiments. Statistical analysis was performed using an unpaired t test. * p<0.05, ** p<0.005, ***p<0.0005 versus binding of ClfB-expressing bacteria.

Initially a streptomycin resistant (Sm^r^) mutant of *S. aureus* Newman and ClfB-deficient mutant Newman Sm^r^ Δ*clfB* (hereafter referred to as Newman and Newman Δ*clfB*) were tested for adhesion to immobilized recombinant human and murine loricrin (GST-human loricrin_1–315_, (Hlor); GST-murine loricrin_1–480_, (MLor)) and to human and murine K10 peptides (GST-human K10_544–563_, (HK10); GST-murine K10_454–570_, (MK10)). *S. aureus* Newman adhered avidly to all ligands (Figure 1). *S. aureus* Newman Δ*clfB* did not adhere detectably to HLor and MLor and exhibited significantly reduced adherence to HK10 and MK10 (Figure 1). This is in agreement with a previous observation that Newman appears to have a second, albeit less potent, K10 adhesin [33]. Complementation of Newman Δ*clfB* with pCU1:*clfB* restored adherence to HLor (Figure S2). These data show that loricrin is a ligand for ClfB.

To establish if ClfB alone can promote bacterial adhesion to immobilized loricrin, adhesion assays were performed using *L. lactis* MG1363 carrying a plasmid that expressed ClfB (pKS80:*clfB*) [40] and compared to *L. lactis* carrying the empty vector. *L. lactis* (pKS80:*clfB*) adhered strongly to each of the proteins indicating that ClfB alone is sufficient for promoting bacterial adhesion to the omega loop-containing ligands (Figure 1).

### Recombinant ClfB N2N3 binds to recombinant human loricrin

**Figure 2:**
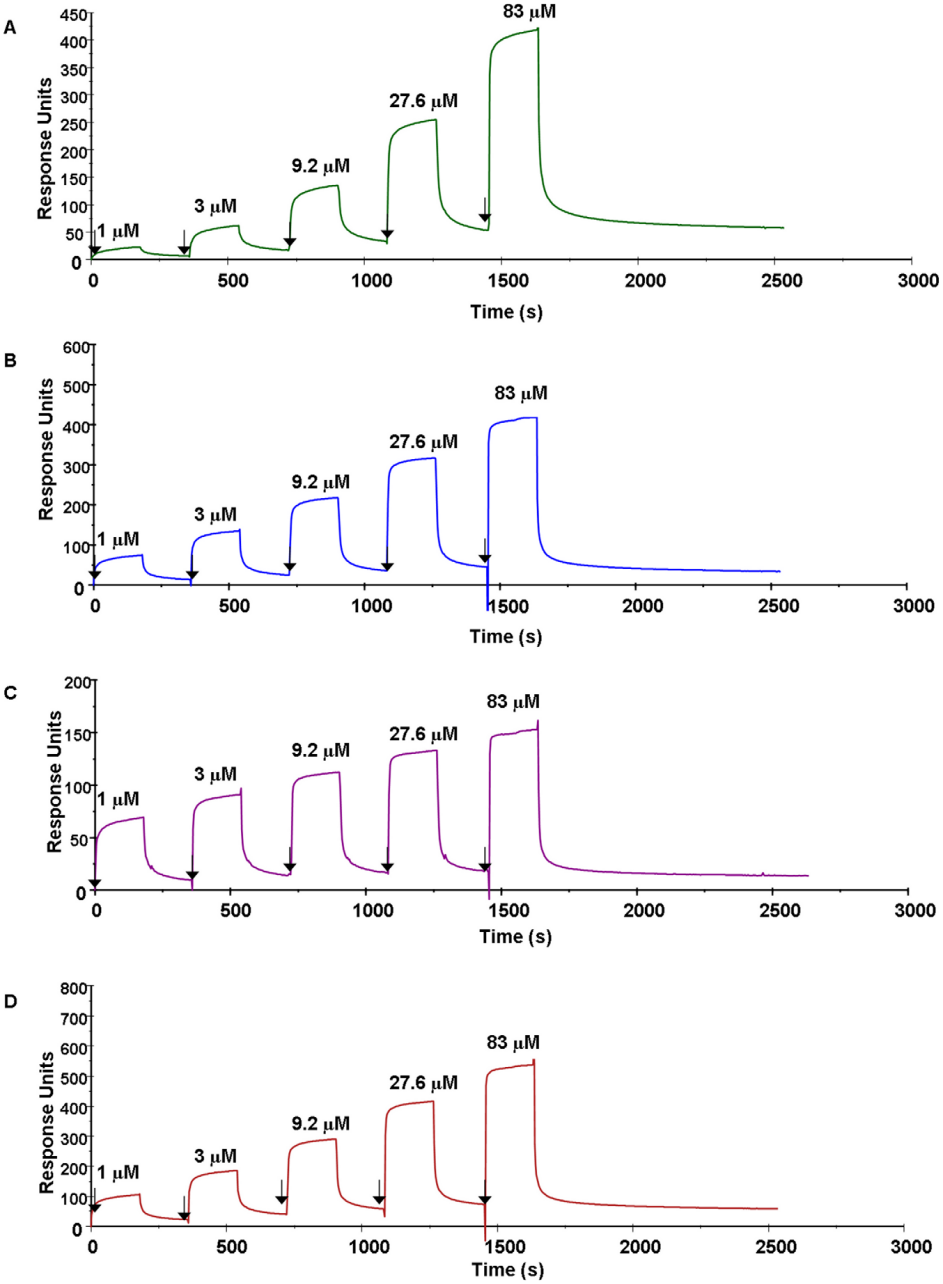
Surface Plasmon Resonance analysis of the interaction of ClfB with loricrin and keratin. Representative sensorgrams display binding of rClfB_201–542_ to and dissocation from (A) GST-HLor, (B) GST-HK10, (C) GST-MLor and (D) GST-MK10 in a single cycle kinetics assay. GST-tagged ligands were captured onto a CM5 chip coated with anti-GST IgG and were exposed to increasing concentrations of rClfB_201–542_. Binding is measured as response units (RU) against time. The affinities were calculated from curve fitting to a plot of the RU values against concentrations of rClfB_201–542_. Arrows indicate the time at which rClfB_201–542_ is injected. The data shown is representative of 3 individual experiments.

In order to demonstrate a direct interaction between ClfB and loricrin and to measure the affinity of binding, surface plasmon resonance (SPR) was employed. HLor was captured on the surface of a sensor chip that had been coated with anti-GST IgG. Previous studies have indicated that the minimum Fg and K10 binding region of ClfB comprises the N2 and N3 subdomains [27]–[28] (Figure S1B). Recombinant ClfB N2N3 (rClfB_201–542_) was expressed with an N-terminal hexahistidine tag. Increasing concentrations of rClfB_201–542_ were passed over the surface of the HLor-coated chip. rClfB_201–542_ bound to HLor in a concentration-dependent manner indicating that the loricrin binding site is located within the ClfB N2N3 subdomains (Figure 2A). From analysis of the equilibrium binding data the dissociation constant (K*_D_*) of the interaction between rClfB_201–542_ and loricrin was determined to be 4.33±1.1 µM. Similar experiments were carried out with MLor (Figure 2B) which had a slightly lower affinity for rClfB_201–542_ (K*_D_* = 15.66±3.4 µM).

The affinity of rClfB_201–542_ for GST-tagged proteins corresponding to high affinity binding sites in human (Figure 2C) and murine (Figure 2D) K10 was determined by the same method. rClfB_201–542_ bound HK10 and MK10 with a similar affinity as it did to HLor and MLor, respectively (Table 1). The affinity of rClfB_201–542_ for a GST-tagged protein corresponding to repeat domain 5 of the αC region of Fg (GST-Fgα_316–367_) was also measured. The K*_D_* measured for GST-Fgα_316–367_ was 5.25 µM, in the same low µM range as the other ligands. This indicates that ClfB has a similar affinity for loricrin, K10 and the αC-region of Fg.

**Table 1:** Affinities of ClfB N2N3_201–542_ for loricrin, keratin and fibrinogen using surface plasmon resonance.

GST-Tagged Protein	K_D_ (µM) ± SE*
HLor	4.33±1.10
MLor	15.66±3.40
HK10	7.89±2.10
MK10	14.38±3.0
Fgα_316–367_	5.25±1.5
Loricrin Loop Region 2v	2.21±1.10
Loricrin Loop Region 2	3.31±0.81
Loricrin Loop Region 3	5.47±1.40
Loricrin Loop Region 1A	16.70±2.30
Loricrin Loop Region 1B	34.48±2.70

* Data representative of n = 3 individual experiments.

SPR was also used to identify binding sites within human loricrin. Loricrin is composed of three Gly-Ser-rich regions capable of forming omega loops (Figure S3) [37]. Regions 2 and 3 are separated by short stretches rich in glutamine, where transglutamination reactions occur [36]. The N- and C-termini of the protein also comprise short Glu-rich stretches. Region 1 contains the largest omega loop region, which is interrupted by a single lysine (K_88_). In order to determine if individual loop regions could bind ClfB, DNA was synthesized corresponding to the individual regions along with flanking sequences (Figure S3A). As with full length loricrin and the K10 sequences, DNA was cloned into a pGEX vector allowing expression and purification of GST-tagged proteins. Region 1 was divided at K_88_ in order to make two separate constructs designated 1A and 1B. Loop region 1A began at residue S_1_ and terminated at residue K_88._ Loop region 1B began at K_88_ and terminated at S_159_. Loop region 2 began at residue G_152_ and terminated at S_230_. One variant of the second loop region (loop region 2v), which corresponds to an allelic variant of the *lor* gene (a 12 base pair deletion) [39] that results in a loop that is 4 residues shorter was also expressed (G_152_-S_226_). Loop region 3 spanned amino acids S_216_-K_315_.

The affinity of ClfB N2N3_201–542_ for loop regions 1A, 1B, 2, 2v and 3 was measured by SPR. Recombinant ClfB N2N3_201–542_ bound all GST-tagged loop regions and the K*_D_* for each interaction was measured (Table 1). ClfB bound loop region 2v and 2 with the highest affinity (*K_D_* = 2.21±1.1 µM and 3.31±0.81 µM respectively). Loop region 1B had the lowest affinity for ClfB (34.48±2.70 µM). These data indicate that loricrin contains more than one binding site for ClfB and that the highest affinity binding site is present within loop region 2.

### ClfB likely binds to loricrin using the “dock, lock and latch” mechanism

**Figure 3:**
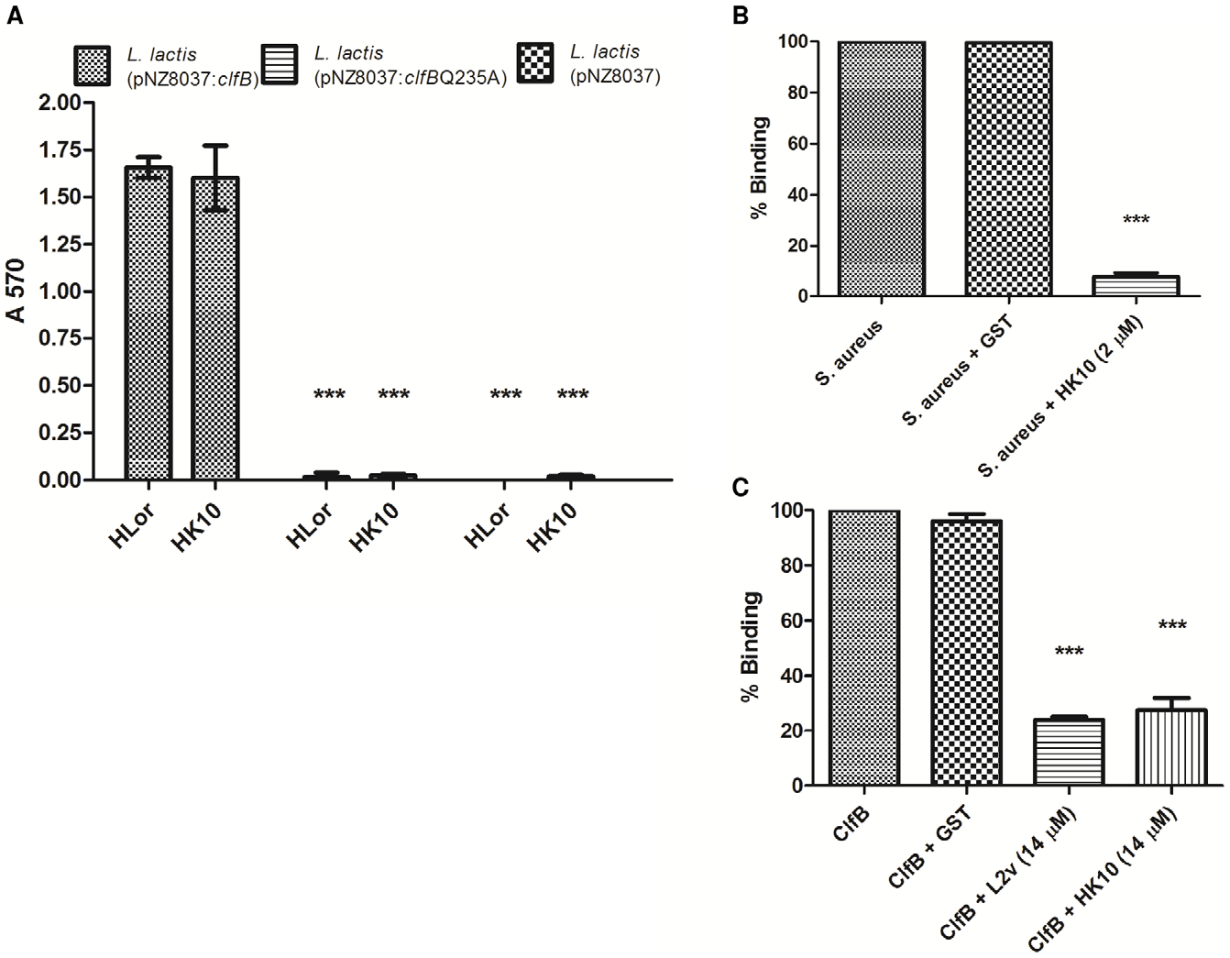
Inhibition of bacterial adherence and rClfB binding to immobilized ligands. (A) *L. lactis* NZ9800 (pNZ8037), *L. lactis* NZ9800 (pNZ8037:*clf*B) and *L. lactis* NZ9800 (pNZ8037:*clf*BQ235A) were added to wells containing immobilized GST-tagged Hlor, MLor, HK10, MK10 (0.5 µM). Bacterial adherence was measured by staining with crystal violet and measurement of the absorbance at 570 nm. The data shown is representative of two individual experiments (B). *S. aureus* Newman pre-incubated with GST or HK10 (2 µM) was added to loricrin-coated microtitre wells (0.5 µM). Bacterial adherence was measured by staining with crystal violet and measurement of the absorbance at 570 nm and was expressed as a percentage of total binding. Values represent the mean ± SD of triplicate wells. The data shown is representative of two individual experiments. (C) Recombinant ClfB N23_201–542_ was pre-incubated with GST, HK10 or L2v (14 µM) before being added to loricrin-coated microtitre wells (0.5 µM). Bound protein was detected using HRP-conjugated anti-his antibodies and was expressed as a percentage of total bound protein. Values represent the mean ± SD of triplicate wells. The values shown are representative of 3 individual experiments. Statistical analysis was performed using an unpaired t-test. *** p<0.0005 versus binding of ClfB-expressing bacteria (A) or pre-incubation with GST (B, C).

ClfB binds to K10 and Fg peptide ligands using the “dock, lock and latch” mechanism [28]–[29]. In order to determine whether ClfB binds loricrin by this mechanism, adhesion assays were performed using *L. lactis* NZ9800 carrying a plasmid which expresses a variant of ClfB (Q235A) that cannot bind Fg or K10 [30], [41]. Glu_235_ makes direct contact with Fg and K10 in the ClfB binding trench and this interaction is crucial for binding to occur by the “dock, lock and latch” mechanism [28]. *L. lactis* expressing ClfB_Q235A_ (*L. lactis* pNZ8037:*clfB*Q235A) did not adhere detectably to HLor or HK10 in comparison to *L. lactis* expressing wild-type ClfB (*L. lactis* pNZ8037:*clfB*) (Figure 3A). When induced with the same concentration of nisin, the expression levels of ClfB and ClfB_Q235A_ on the surface of *L. lactis* were equal (data not shown). This suggests that ClfB may bind HLor using the “dock, lock and latch” mechanism.

Recombinant HLor loop region L2v and HK10 were used in inhibition studies in order to provide further evidence that ClfB uses the “dock, lock and latch” mechanism to bind loricrin. Pre-incubation of *S. aureus* with HK10 almost completely abolished its ability to bind immobilised loricrin (Figure 3B). Similarly, pre-incubation of recombinant ClfB with HK10 (or L2v) inhibited its ability to bind immobilised loricrin (Figure 3C). These data indicate that HLor and HK10 bind to the same or overlapping sites in ClfB, providing strong evidence that loricrin is also bound using the “dock, lock and latch” mechanism.

### *S. aureus* binding to human squamous epithelial cells is dependent on an interaction between ClfB and loricrin

**Figure 4:**
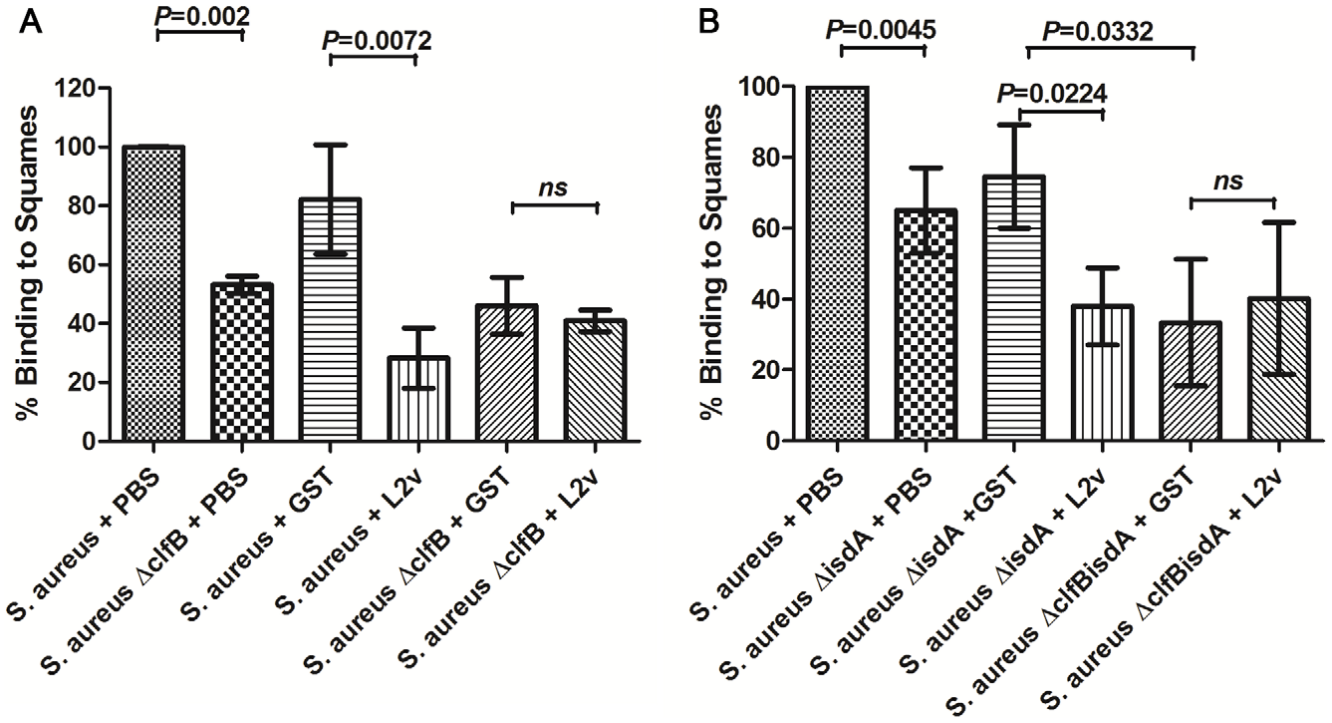
HLor region L2v blocks ClfB-mediated adherence of *S. aureus* to human desquamated epithelial cells. *S. aureus* strains were grown to exponential phase in TSB (A) or in RPMI (B). Washed cells were incubated with recombinant GST or recombinant L2v-GST, or just resuspended in PBS, before being incubated with human nasal epithelial cells. Adherent bacteria were enumerated by microscopy and were expressed as a percentage of the positive control. Results are expressed as the mean ± SD of 3 independent experiments. Statistical analysis was performed using an unpaired t test.

ClfB has previously been shown to facilitate adherence of *S. aureus* to squames [19], [22]. In order to determine whether loricrin plays a major role in ClfB-mediated *S. aureus* adherence to squames, *S. aureus* was pre-incubated with recombinant loricrin loop region L2v and was then tested for adherence to nasal squamous epithelial cells. Pre-incubation of *S. aureus* with L2v caused a significant (p = 0.0072) reduction in adherence to squames compared to *S. aureus* pre-incubated with GST alone (Figure 4A). This illustrates that an interaction with loricrin is necessary for efficient *S. aureus* adherence to squames. Consistent with previously published studies [19], [22], adherence of *S. aureus* Δ*clfB* to squames was also significantly reduced (p = 0.002). However, there was no further reduction in adherence when *S. aureus* Δ*clfB* was pre-incubated with L2v compared to GST alone, indicating ClfB is the only *S. aureus* factor binding to loricrin on squames (Figure 4A).

IsdA has been shown to play a role in *S. aureus* adherence to squames under iron-limited conditions, and is also known to facilitate adherence to recombinant human loricrin *in vitro* [22], [42]. To establish if an interaction between IsdA and loricrin occurs during adherence to squames, squamous cell adherence assays were repeated using *S. aureus* grown under iron-limited conditions. Consistent with our previous results (Figure 4A), we saw a similar significant reduction in adherence to squames by *S. aureus* Δ*clfB* and by wild-type *S. aureus* pre-incubated with L2v, with no further reduction in adherence when *S. aureus* Δ*clfB* was pre-incubated with L2v compared to GST alone (Figure S4). These data confirm that even under conditions in which IsdA is maximally expressed L2v is only inhibiting the interaction between ClfB and squames.

To confirm that IsdA plays a role in *S. aureus* adherence to human squames we studied a Δ*isdA* mutant of Newman, with bacteria grown in iron-limited conditions. There was a significant reduction (p = 0.0045) in adherence of *S. aureus* Δ*isdA* to squames when compared to a wild-type strain (Figure 4B). Pre-incubation of *S. aureus* Δ*isdA* with L2v significantly (p = 0.0224) impaired adherence to squames compared to pre-incubation with GST alone, most likely by inhibiting the interaction between ClfB and its ligand(s).

A double mutant (*S. aureus* Newman Δ*clfB*Δ*isdA*) had a similar impaired ability to adhere to squames, to that observed when *S. aureus* Δ*isdA* was pre-incubated with L2v (p = 0.0322). Pre-incubation with L2v did not cause any further reduction in adherence of *S. aureus* Δ*clfB*Δ*isdA* (Figure 4B). Taken together, these results confirm that IsdA contributes significantly to adherence of *S. aureus* to human squames but that this does not involve an interaction between IsdA and loricrin loop region L2v.

### *S. aureus* nasal colonisation is impaired in loricrin-deficient mice

Having identified loricrin as a ligand for ClfB *in vitro*, the importance of loricrin in *S. aureus* nasal colonisation was then investigated. Nasal colonisation was established in specific pathogen-free wild-type FVB (WT) mice and loricrin-deficient mice (Lor^−/−^) on the same background. To establish colonisation, mice were inoculated intra-nasally with *S. aureus* Newman Sm^r^ (2×10^8^ CFU) (hereafter called Newman). Mice were administered streptomycin in their drinking water 24 hours prior to inoculation and for the duration of the experiment in order to reduce interference from the commensal bacterial flora. At specific time points after inoculation, nasal tissue was excised and homogenized and the number of CFU per nose enumerated.

**Figure 5:**
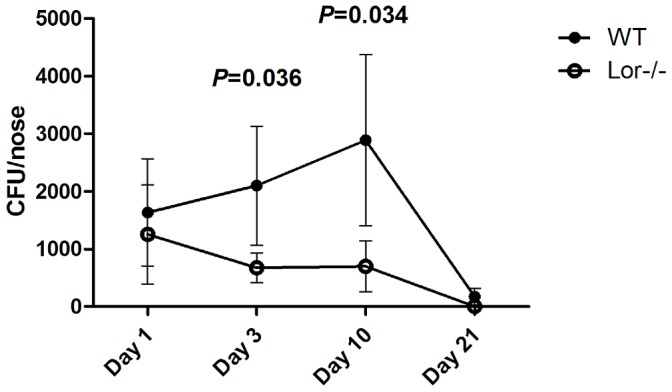
Nasal colonisation of *S. aureus* in the FVB wild-type and Lor^−/−^ mouse. Mice were inoculated intra-nasally with *S. aureus* Newman (2×10^8^ CFU). Mice were euthanized and bacterial burden in the noses established on days 1, 3, 10 and 21. Results expressed as mean Log CFU per nose (n = 15, per group). Statistical analysis was performed using the Mann-Whitney test.

On day 1, WT and Lor^−/−^ mice had similar levels of Newman in their noses. WT mice remained stably colonised with Newman over a period of 10 days with the number of bacteria actually increasing slightly during this period. This suggested that the bacteria had adhered to the nasal epithelium and were able to proliferate. In contrast, there was a significant reduction in the levels of Newman present in the noses of Lor^−/−^ mice compared to WT mice on day 3 (*p* = 0.0355) and day 10 (*p* = 0.0343) indicating that Lor^−/−^ mice were unable to retain *S. aureus* in their noses (Figure 5, Figure S5A). By day 21, Newman was completely cleared from the noses of Lor^−/−^ mice, while low numbers of bacteria were still detectable in the WT mice. These results suggest that the absence of loricrin does not impact initial attachment of *S. aureus* to the nasal tissue, but the protein appears to be essential for the maintenance of the bacterium in the nose and for sustained colonisation up to a period of 21 days. No bacteria were detectable in the blood of either WT or Lor^−/−^ mice (data not shown) and similar low levels of bacteria were detectable in the lungs of WT and Lor^−/−^ at 3 days (median = 1 CFU) and 10 days (median<10 CFU) post inoculation, indicating that minimal systemic dissemination of the bacteria occurred.

To confirm the role played by loricrin in mediating nasal colonisation by *S. aureus*, we investigated the ability of recombinant loricrin to inhibit colonisation in WT mice. Mice were inoculated intra-nasally with *S. aureus* Newman in combination with recombinant L2v or GST on day 1 and day 2 post inoculation. Nasal colonisation with Newman was significantly decreased on day 3 in the presence of recombinant loricrin but not GST (Figure S5B).

To investigate if the loricrin defect also affected invasive infection, groups of WT and Lor^−/−^ mice were challenged by intra-peritoneal injection with *S. aureus* Newman (5×10^8^ CFU). On day 2-post infection there were no significant differences in the levels of systemic bacterial infection between the WT and Lor^−/−^ mice (Table 2). These data suggest that the interaction that is occurring between *S. aureus* and loricrin is specific to the nasal passage.

**Table 2:** Systemic infection in Lor^−/−^ mice.

	Peritoneal Cavity	Blood	Liver	Kidney	Spleen
WT	3.26±0.52	5.46 ±0.27	7.24 ±0.11	5.01±0.12	5.4±0.41
Lor^−/−^	3.59±1.31	6.24±0.53	6.91±0.30	5.42±0.90	6.25±0.65
	p = 0.8571	p = 0.2286	p = 0.6286	p = 0.6286	p = 1

Results expressed as mean Log CFU/ml of fluid or homogenised tissue ± SEM, n = 4 per group.

### Nasal expression of loricrin and cytokeratin is not affected by *S. aureus* nasal colonisation

**Figure 6:**
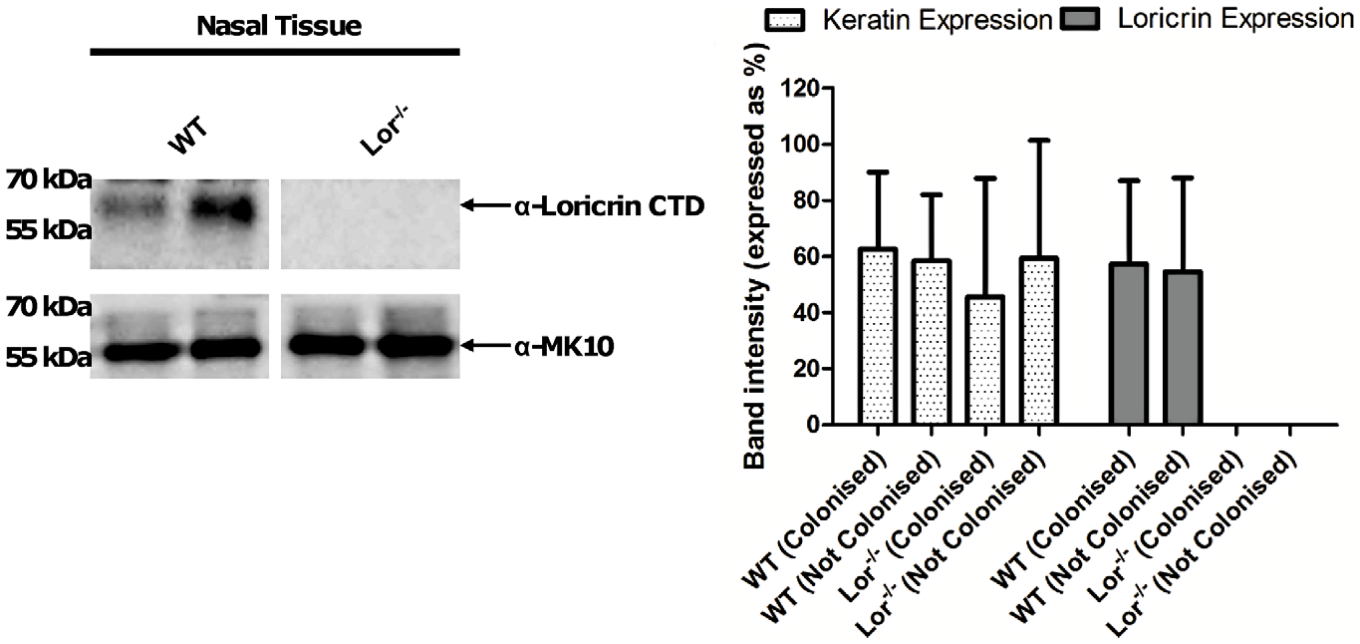
Expression of loricrin and cytokeratin during *S. aureus* nasal colonisation. Nasal tissue from WT and Lor^−/−^ mice was excised and homogenised in PBS. Soluble proteins were extracted and analysed by Western immunoblotting using rabbit anti-murine loricrin IgG followed by HRP-conjugated goat anti-rabbit IgG. Bound antibody was removed and the filter was re-probed with rabbit anti-murine K10 IgG followed by HRP-conjugated protein A. Band intensity was measured using ImageQuant software and was expressed as a percentage of the highest intensity band. Data represents mean ± SD, n = 4 mice, per group.

In order to investigate any variability in expression of loricrin and K10 that occurred during nasal colonisation with *S. aureus*, loricrin and keratin expression in the noses of FVB and Lor^−/−^ mice was compared in the absence of *S. aureus* nasal colonisation and on day 10 post colonisation. Nasal tissue was excised from WT and Lor^−/−^ mice. Proteins were solubilised from nasal tissue homogenates, separated by SDS-PAGE and Western immunoblotting performed, probing with loricrin-specific antibodies. A ∼56 kDa band corresponding to loricrin was seen in the nasal tissue from WT but not Lor^−/−^ mice (Figure 6). Consistent with previously published data [20], there was no detectable difference in K10 expression in Lor^−/−^ mice when compared to WT mice, either in the absence or presence of *S. aureus* nasal colonisation (Figure 6). Similarly the levels of loricrin expression did not vary significantly in colonised or non-colonised animals. We did observe some variation in the levels of loricrin in the nasal tissue of individual mice, however this variation did not correlate with the variability seen in *S. aureus* colonisation levels in these mice (data not shown).

### Expression of ClfB *by L. lactis* facilitated nasal colonisation in WT but not Lor^−/−^ mice

**Figure 7:**
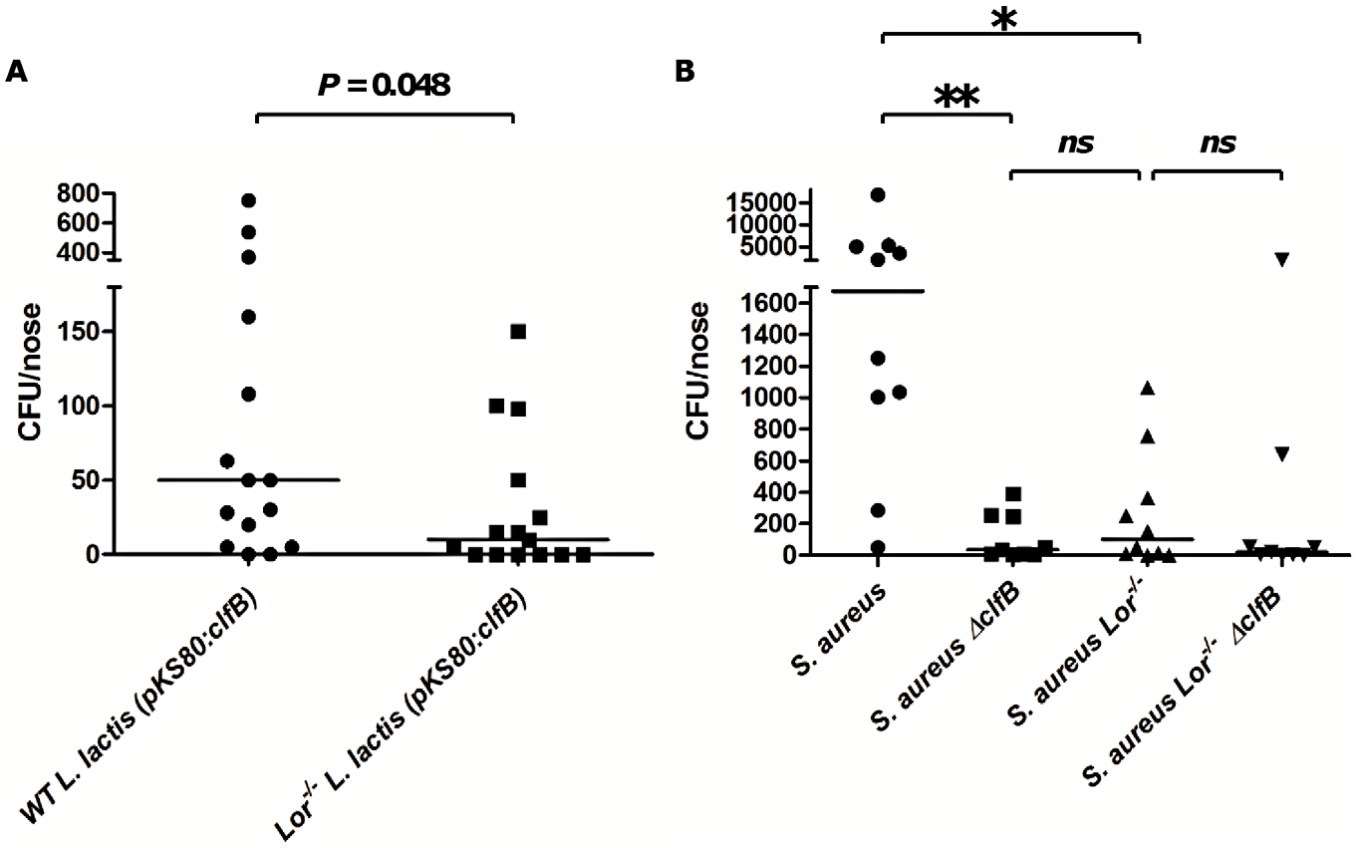
Nasal colonisation of *L. lactis* expressing ClfB and Newman Δ*clfB* ^−^ in the FVB wild-type and Lor^−/−^ mice. (A) Mice were inoculated intra-nasally with *L. lactis* MG1363 (pKS80) or *L. lactis* MG1363 (pKS80:*clf*B) (2×10^11^ CFU). Mice were euthanized after 24 hours and the bacterial burden in noses was established. Inoculation with the empty vector (pKS80) did not result in significant colonisation (>5 CFU per nose) in either WT or Lor^−/−^ mice. Statistical analysis was performed using the Mann-Whitney test. (B) Mice were inoculated intra-nasally with Newman or Newman Δ*clfB* (2×10^8^ CFU). After 10 days, mice were euthanized and bacterial burden in the noses was established. Each dot indicates the number of CFU/nose for a single mouse. Results expressed as Log CFU per nose, median indicated by bar (n = 15–20 per group). Statistical analysis was performed using the Krustal-wallis test and Dunns Multiple Comparisons test. *p<0.05, **p<0.005.

To investigate further the importance of ClfB in mediating the interaction between *S. aureus* and loricrin during colonisation, we developed a novel model of nasal colonisation using *L. lactis* expressing ClfB. Groups of WT and Lor^−/−^ mice were inoculated intra-nasally with 2×10^11^ CFU *L. lactis* (pKS80:*clfB*) or *L. lactis* (pKS80) as a control. After 24 hours, significant levels of ClfB-expressing *L. lactis* could be recovered from the noses of WT mice (Figure 7A), while there was a ∼80% reduction in the levels of ClfB-expressing *L. lactis* colonizing the noses of Lor^−/−^ mice. The majority of mice did not retain the control strain *L. lactis* (pKS80) in their noses (>5 CFU). These results demonstrate that the interaction between ClfB and loricrin is sufficient to facilitate nasal colonisation. *L. lactis* was not detected in the lungs of either mouse strain (data not shown).

### A ClfB^−^ mutant of *S. aureus* Newman exhibited reduced nasal colonisation in WT but not Lor^−/−^ mice

To confirm the importance of the interaction between ClfB and loricrin in *S. aureus* nasal colonisation, groups of WT and Lor^−/−^ mice were inoculated intra-nasally with Newman or Newman Δ*clfB*. After 10 days, the nasal bacterial burden was quantified. There was a significant reduction (*p* = 0.015) in colonisation of WT mice by Newman Δ*clfB* compared to the parental strain (Figure 7B), confirming the role played by ClfB in nasal colonisation. In contrast, there was no significant difference between colonisation with the parental Newman strain and Newman Δ*clfB* in the Lor^−/−^ mice. By day 10 both Newman and Newman Δ*clfB* were almost completely cleared from the nares of Lor^−/−^ mice. To confirm the importance of the ClfB-loricrin interaction we generated a *clfB* mutant of strain SH1000. Similar results were obtained when we performed colonisation experiments using this strain (Figure S6). From these data we can conclude that loricrin is the primary ligand for ClfB *in vivo* and is required for the maintenance of *S. aureus* during nasal colonisation.

## Discussion

It is well established that nasal carriage of *S. aureus* represents a significant risk factor for subsequent infection with this organism [2]–[4]. Current strategies for decolonising carriers rely on the use of topical treatment with the antibiotic mupirocin to which *S. aureus* is becoming increasingly resistant [5], [43]. The development of new therapeutic options for controlling nasal colonisation by this organism requires a deeper appreciation of the molecular interactions that occur between the bacterium and the host at the nasal epithelial surface. In this study, we demonstrate for the first time that nasal colonisation with *S. aureus* is critically dependent upon an interaction between the squamous epithelial cell cornified envelope protein loricrin and the *S. aureus* surface protein ClfB.

Previous studies have identified an important role for ClfB in *S. aureus* nasal colonisation [23]–[24], and have demonstrated that ClfB can promote adhesion to squames *in vitro* [19], [33]. Given that the dominant CE protein loricrin is composed of GS-rich omega loops [14]–[16], we predicted that this protein might be an important target for ClfB binding *in vivo* during *S. aureus* nasal colonisation.

Using bacterial adherence assays we have demonstrated that ClfB promotes adherence of *S. aureus* to immobilized loricrin. *S. aureus* Newman grown to exponential phase in TSB adhered strongly to loricrin whereas bacteria lacking ClfB did not adhere. Consistent with previous findings we demonstrated that ClfB also promotes adherence of *S. aureus* to cytokeratin 10. Furthermore, ClfB promoted adherence of *L. lactis* to immobilized loricrin. Taken together these results indicate that *S. aureus* adhesion to loricrin is dependent on the expression of ClfB.

We used SPR to demonstrate a direct interaction between recombinant ClfB and loricrin and to measure the affinity of binding. The loricrin binding site is located in the N2N3 subdomains of ClfB. ClfB bound to human loricrin with a *K_D_* of 4.33±1.10 µM, which is similar to the affinities for the ClfB-K10 and ClfB-Fg interactions (7.89±2.10 and 5.52±1.5 µM respectively). The *K_D_* determined here for rClfB_201–542_ binding to GST-HK10 by SPR (7.89±2.10 µM) is similar to the *K_D_* previously determined for rClfB binding to His-tagged rMK10_454–570_ and synthetic HK10 peptides in solution (isothermal titration calorimetry, 1.4 µM, intrinsic tryptophan fluorescence, 1.7 and 5.4 µM, respectively [33]).

By subdividing the human loricrin molecule into three major loop regions we demonstrated that binding sites for ClfB exist throughout the protein. However the highest affinity ClfB binding site was localised to loop region 2. Previous studies on the human loricrin gene have shown that the major loop region designated loop region 2 contains a polymorphism, and can undergo a 12 bp deletion, resulting in a loop region that is 4 amino acids shorter [38]–[39]. We synthesized two size variants of loop region 2 in order to investigate whether this particular polymorphism had an effect on the binding ability of ClfB. The results from SPR analysis showed that the affinities of ClfB for both loop 2 variants are similar. Nevertheless, it is possible that other sequence variants of loricrin may have an effect on the ability of ClfB to bind.

The ability of ClfB to recognise murine K10 and loricrin was also tested. ClfB promoted bacterial adherence to MK10 and loricrin in a similar way to the human proteins and rClfB bound to MK10 and loricrin similarly to HK10 and loricrin, albeit with a slightly reduced affinity. There are size and sequence differences between human and murine loricrins, but they have similar amino acid composition and omega loop region organization [37] (Figure S3). In addition, it has been shown using fluorescence spectrometry and circular dichroism that the structures of recombinant human loricrin and murine loricrin are indistinguishable in solution [36]. We can therefore assume with confidence that the ClfB-loricrin interactions that were characterised *in vitro* would have *in vivo* relevance in our murine nasal colonisation model.

Previous studies demonstrated that ClfB containing amino-acid substitution Q235A is defective in K10- and Fg-binding by the “dock, lock and latch” mechanism [28], [30], [41]. Residue Q235 is located in the ligand binding trench and makes direct contact with the K10 and the Fg peptide [28]. *L. lactis* expressing ClfB_Q235A_ was unable to adhere to loricrin. Furthermore, pre-incubation of *S. aureus* cells or recombinant ClfB with HK10 inhibited binding to loricrin, indicating that both ligands recognise similar sites in ClfB. This is consistent with ClfB binding to loricrin by the “dock, lock and latch” mechanism. This is supported by the similarities between the sequences recognised by ClfB in K10 and Fg (GSSG*X*G motif) [28] and the GS-rich regions in loricrin. Solving the crystal structure of ClfB in complex with peptides corresponding to binding sites within loricrin will provide further insight into the mechanism of loricrin binding by ClfB.

It is clear that nasal colonisation with *S. aureus* is a multifaceted process that involves the interaction of several bacterial surface molecules with different host ligands [25]. However, we have shown that a specific interaction between ClfB and loricrin is critically important for the adherence of *S. aureus* to human squames. In agreement with previous studies, adherence to squames was not completely abolished in the absence of ClfB which is consistent with this being a multifactorial process. We did not observe any reduction in adherence to squames after pre-incubating *S. aureus* Δ*clfB* with L2v, indicating that the loop region of loricrin is not bound detectably by other staphylococcal surface proteins under these conditions. Furthermore the *in vivo* studies demonstrate that in the absence of loricrin, *S. aureus* nasal colonisation is reduced by approximately 80% confirming the absolute requirement of loricrin for this process. Interestingly however, the absence of loricrin during systemic infection does not appear to impact upon bacterial dissemination. Both WT and Lor^−/−^ mice were initially colonised with *S. aureus* to the same extent suggesting that the loricrin-ClfB interaction is not required for initial attachment of bacteria to the nasal tissue. However over time the Lor^−/−^ mice were unable to retain *S. aureus* in their noses compared to the WT animals. A low level of *S. aureus* colonisation was achieved in the Lor^−/−^ mice, presumably due to the ability of ClfB and/or other *S. aureus* factors to bind alternative receptors [19], [33], [42].

Translating our *in vitro* observations to the *in vivo* situation has been difficult. *S. aureus* is a human commensal and does not normally colonise rodents, so a major challenge for the field is to establish robust and sustained levels of nasal colonisation with *S. aureus* in rodents. Nasal colonisation of mice is particularly attractive due to the availability of transgenic and knock-out animals which facilitate in-depth investigations into the interaction of the commensal organism with the host. The availability of loricrin-deficient animals has afforded us a unique opportunity to characterise for the first time *in vivo*, a specific interaction between a host and a bacterial factor that facilitates the process of nasal colonisation. *S. aureus* nasal colonisation in mice may be influenced by factors such as mouse strain, bacterial strain and bacterial load [23], [44]. Using an inbred strain of mouse, we achieved a stable level (≤10^3^ CFU per nose) of nasal colonisation for a period of 21 days that were comparable to those previously observed in outbred mouse strains [44].

The specificity of the interaction between ClfB and loricrin *in vivo* was established using a novel murine nasal colonisation model in which mice were inoculated with the surrogate host *L. lactis* expressing ClfB. These studies demonstrated that expression of ClfB alone is sufficient to promote nasal colonisation, without any dependence on other staphylococcal factors. The low numbers of bacteria recovered from the noses of these mice and the short duration of colonisation is likely due to the fact that *L. lactis* is an avirulent and nutritionally fastidious organism [45] that grows optimally at 28–30°C. We observed an 80% decrease in the levels of *L. lactis* ClfB^+^ colonisation in Lor^−/−^ mice compared to WT mice confirming that loricrin represents the major binding target for ClfB in the nares. The residual binding of *L. lactis* ClfB^+^ likely reflects a minimal interaction of ClfB with other CE proteins such as K10. A similar model using a K10-deficient mouse [46] would be required to establish definitively the relative contribution of these two CE proteins to colonisation.

Although redundancy occurs in *S. aureus* surface-expressed factors that promote binding to host squames, our data indicate that the interaction between ClfB and loricrin is crucial for *S. aureus* nasal colonisation. Consistent with previous studies we demonstrated that a ClfB-deficient mutant of *S. aureus* was significantly impaired in its ability to colonise WT mice compared to the parental *S. aureus* strain. In contrast, a similar low level of colonisation was achieved when Lor^−/−^ mice were inoculated with either the *clfB* mutant or the parental strain confirming the specificity of the interaction between ClfB and loricrin *in vivo* during *S. aureus* nasal colonisation.

It has been assumed that the interaction between ClfB and K10 is crucial for nasal colonisation by *S. aureus* [19], [33]. However, our data demonstrates that loricrin is recognised by ClfB *in vivo* and suggests that K10 may not serve as its major ligand in the nose. IsdA is the only other cell-wall anchored surface protein with a proven role in *S. aureus* nasal colonisation [22]. *In vitro* studies have shown that IsdA promotes bacterial adhesion to components of the CE such as loricrin, involucrin and K10 [42]. Consistent with this we demonstrated reduced adherence to human squames by *S. aureus* Δ*isdA*. However, our data implies that IsdA does not interact with loricrin loop region 2v on human squames. Pre-incubation of *S. aureus* Δ*isdA* but not *S. aureus* Δ*clfB*Δ*isdA* with L2v resulted in a reduction in adhesion to squames suggesting that in the absence of IsdA there are other ligands to which loricrin can bind and subsequently inhibit squame binding, whereas in the absence of ClfB this is not the case.

A mutant of *S. aureus* lacking wall teichoic acid (WTA) was also shown to be defective in its ability to colonise the nares of cotton rats after only one day suggesting that WTA-mediated attachment may also be important for the initial stages of colonisation [47]. In contrast an IsdA-deficient mutant of *S. aureus* had reduced colonisation ability over an extended time course [22] similar to the results obtained for a ClfB-deficient mutant in this study and by others [23]. This data suggests that different molecules expressed by *S. aureus* may have distinct roles to play in facilitating nasal colonisation. Further studies, that are beyond the scope of this paper, are required to determine the nature of their relative contributions to *S. aureus* nasal colonisation *in vivo*.

The primary finding from our study is that loricrin is the major binding partner for ClfB during *S. aureus* nasal colonisation. The bacterial adherence data revealed that loricrin is a ligand for *S. aureus* and that this interaction is facilitated specifically by ClfB. SPR analysis confirmed that ClfB binds the omega loop regions of loricrin and provides new information on the relative affinities that ClfB has for its ligands. Similar to previous rodent studies, our mouse models have proven useful in the characterization of factors involved in nasal colonisation. Our *in vivo* findings have confirmed that ClfB is one of the primary bacterial adhesins involved in nasal colonisation and have provided further *in vivo* evidence that ClfB is a crucial promoter and mediator of *S. aureus* carriage in the nose. Furthermore, through the use of a gene-deficient mouse model we have defined the mechanism by which ClfB interacts with the host, through binding to the CE protein loricrin. We can conclude therefore that loricrin is a major determinant of *S. aureus* nasal colonisation and represents the most important target for ClfB in the nose.

## Materials and Methods

### Ethics statement

Experiments on mice were conducted under Irish Department of Health guidelines with ethical approval from the Trinity College Dublin ethics committee. Ethical approval for the use of human squames was obtained from the TCD Faculty of Health Sciences ethics committee.

### Mice

Female FVB mice were obtained from Harlan UK. Loricrin-deficient FVB mice have been previously described [20] and were obtained from Dr. Dennis Roop, University of Colorado Anschutz Medical Centre, Colorado, USA and were bred in-house at Trinity College, Dublin.

### Bacterial strains and growth conditions

*E. coli* strains XL1-Blue (Stratagene) and DNA cytosine methyltransferase mutant DC10B [48] were used as hosts for cloning. They were grown with shaking in L broth or on L agar at 37°C. *S. aureus* Newman strains were grown to exponential or stationary phase with shaking in tryptic soy (TS) broth or on TS agar at 37°C. RPMI 1640 medium (Sigma) was used to grow bacteria under iron-limitation. *S. aureus* SH1000 strains were grown on TS agar at 37°C. *L. lactis* MG1363 (pKS80) [40], *L. lactis* MG1363 (pKS80:*clf*B) [40], *L. lactis* NZ9800 (pNZ8037) [49], *L. lactis* NZ9800 (pNZ8037:*clf*B) [41] and *L. lactis* NZ9800 (pNZ8037:*clf*BQ235A) [41] were grown statically in brain heart infusion (BHI) broth or agar at 28°C. Nisin (150 ng/ml, Sigma) was added to *L. lactis* NZ9800 cultures to induce expression of ClfB. Antibiotics were added to the media as required: ampicillin (100 µg/ml), streptomycin (500 µg/ml), chloramphenicol (10 µg/ml) and erythromycin (10 µg/ml).

### Strain construction

A streptomycin-resistant mutant of *S. aureus* strain Newman (Newman Sm^r^) and *S. aureus* strain SH1000 was isolated by growth overnight in TSB, and then plating onto TSA plates containing streptomycin (500 µg/ml). Mutations in the *rpsL* gene often result in high level streptomycin resistance [50]–[51]. The *rpsL* gene was amplified by PCR from Newman and Newman Sm^r^ DNA. DNA sequencing revealed a single nucleotide substitution that resulted in a single site amino acid substitution (K55T) in the S12 protein of 30S ribosomal subunit. Newman Sm^r^ was phenotypically identical to its parent strain in terms of growth rate, expression of ClfB (Figure S4) and haemolysis on sheep blood agar (data not shown) [52]–[53].

*S. aureus* Newman Sm^r^ Δ*clfB* and *S. aureus* SH1000 Sm^r^ Δ*clfB were* constructed by allelic exchange using pIMAY [48]. Briefly, primers were designed to amplify 500 bp of DNA located upstream and downstream of *clfB* (Table S1) to delete the entire gene leaving only the start and stop codons. Genomic DNA from Newman or SH1000 was used as template and the resulting PCR products were denatured, allowed to reanneal via the complementary sequences in primers B and C and then amplified using primers A and D, resulting in a 1000 bp fragment consisting of linked sequences upstream and downstream of the *clfB* gene (Δ*clfB* cassette). The amplimer was cloned into pIMAY between EcoRI and SalI restriction sites. The plasmid was transformed into *E. coli* DC10B [48] and then transformed into electrocompetent Newman Sm^r^ or SH1000 Sm^r^. Deletion of the *clfB* gene was achieved by allelic exchange [48]. The resulting ClfB-deficient strains (Newman Δ*clfB*, SH1000 Δ*clfB*) were phenotypically identical to their parent strains in terms of growth rate and haemolysis on sheep blood agar [52]–[53]. Lack of expression of ClfB in *S. aureus* Newman was verified by Western immunoblotting (Figure S7).

*S. aureus* Newman Δ*clfB* was complemented with pCU1 carrying the full length *clfB* gene [26]. Plasmid pCU1:*clfB* was transformed into *E. coli* DC10B [48] and then transferred to Newman Δ*clfB*.

IsdA mutants of *S. aureus* Newman and *S. aureus* Newman Δ*clfB* were constructed using the same method, with primers designed to amplify 500 bp of DNA located upstream and downstream of *isdA* (Table S1).

### SDS-PAGE and Western Immunoblotting

Cell wall-associated proteins were prepared as previously described [54]. Exponential phase cultures were harvested, washed in phosphate-buffered saline and resuspended to OD_600_ of 10 in lysis buffer (50 mM Tris-HCl, 20 mM MgCl_2_, pH 7.5) supplemented with 30% (w/v) raffinose and complete protease inhibitors (40 µl/ml, Roche). Cell-wall proteins were solubilised by incubation with lysostaphin (200 µg/ml; AMBI, New York) for 10 min at 37°C. Protoplasts were removed by centrifugation at 12,000× *g* for 10 min and the supernatant containing solubilised cell-wall proteins was aspirated.

Solubilised cell wall proteins or purified recombinant proteins were boiled for 5 min in Laemmli final sample buffer (Sigma), separated on polyacrylamide gels and transferred onto polyvinylidene difluoride membranes (Roche). Filters were blocked in 10% (w/v) skimmed milk proteins before being probed with antibody. Reactive bands were visualised using the LumiGLO reagent and peroxide detection system (Cell Signalling Technology).

### Antibodies

The following antibodies were used: rabbit anti-murine loricrin IgG (1∶1000; Covance), rabbit anti-murine K10 IgG raised against recombinant full length murine cytokeratin 10_1–570_ (1∶500; Bioresources Unit, Trinity College Dublin), rabbit anti-ClfB A region IgG (1∶1000; described previously [26]) and HRP-conjugated rabbit anti-His antibodies (1∶500, Roche) were used as primary antibodies. HRP-conjugated goat anti-rabbit IgG (1∶3000; Dako) and HRP-conjugated protein A (1∶500; Sigma) were used as secondary labelling reagents.

### Recombinant DNA manipulation and protein expression

Plasmid pCU1:*clfB* [26] was used as template for PCR amplification of DNA encoding amino acids 201–542 of ClfB. Primers incorporating BamHI and HindIII restriction sites, respectively, were used (Table S1). The PCR product was cloned into pCR-blunt-II-TOPO (Invitrogen) and subcloned into pQE30 (Qiagen) which had been cut with BamHI and HindIII. rClfB_201–542_ was expressed and purified from *E. coli* XL-1 Blue by Ni^2+^ affinity chromatography. The protein was analysed by SDS-PAGE and Western immunoblotting.

DNA encoding full length human and murine loricrin, a human K10 peptide, a murine K10 peptide and human loricrin subdomains (1A, 1B, 2, 2v, 3) was codon optimised for *E. coli* and synthesised commercially (Genscript Corporation). DNA was subcloned between the BamHI and EcoRI sites of the expression vector pGEX-4T (GE Lifesciences). DNA encoding human loricrin subdomain 2 (Table S1) was amplified by PCR using plasmid pET11a carrying the full length cDNA clone encoding human loricrin [36] as template and the PCR product was cloned into pGEX-4T2 between BamHI and EcoRI sites. GST-tagged proteins were purified on a GSTrap FF purification column (GE Healthcare) according to the manufacturer's instructions (Figure S3C–E).

### Adherence of bacterial cells to immobilized recombinant proteins

Microtiter plates (Nunc) were coated with recombinant protein (1 µM) in carbonate buffer and incubated overnight at 4°C. Wells were blocked with 5% (w/v) bovine serum albumin (BSA) for 2 h at 37°C. The plates were washed three times with PBS. A bacterial cell suspension (OD_600_ = 1.0 in PBS) was added (100 µl per well), and the plates were incubated for 2 h at 37°C, washed three times with PBS, and bound cells were fixed with formaldehyde (25% v/v) for 20 min and stained with crystal violet (0.5% v/v, 100 µl per well) for 1 min followed by PBS and acetic acid (5% v/v) washes. The absorbance was measured at 570 nm in an ELISA plate reader.

### Inhibition of *S. aureus* and rClfB binding to immobilized human loricrin

*S. aureus* (1×10^8^ colony-forming units) was pre-incubated with recombinant GST, HK10 or Loricrin L2v (2 µM) in PBS at room temperature for 30 min. The bacteria were added to loricrin-coated microtiter wells for 90 min at room temperature. Adherent cells were stained with crystal violet, and the absorbance was measured at 570 nm as described above.

For inhibition studies with recombinant ClfB, loricrin-coated microtitre plates were prepared as above and were blocked with 5% skimmed milk proteins in PBS at 37°C for 2 h. Recombinant ClfB N23_201–542_ (3 µM) was pre-incubated with recombinant GST, HK10 or Loricrin L2v (14 µM) in PBS at room temperature for 1 h. The protein mixture was added to loricrin-coated microtitre wells and was incubated for 1 h at 37°C. Any unbound protein was removed by washing with PBS, and plates were incubated with HRP-conjugated rabbit anti-His antibodies diluted 1∶500 in 1% skimmed milk/PBS for 1 h at room temperature with shaking. 100 µl of a chromogenic substrate solution (1 mg/ml tetramethylbenzidine and 0.006% H_2_0_2_ in 0.05 M phosphate citrate buffer pH 5.0) was added, and plates were developed for 10 min in the dark. The reaction was stopped by the addition of 2 M H_2_S0_4_ (50 µl/well), and plates were read at 450 nm.

### Surface Plasmon Resonance

Surface plasmon resonance (SPR) was performed using the BIAcore ×100 system (GE Healthcare). Goat anti-GST IgG (30 µg/ml; GE Healthcare) was diluted in 10 mM sodium acetate buffer (pH 5.0) and immobilized on CM5 sensor chips using amine coupling as described by the manufacturer. Recombinant GST-tagged protein (10–30 µg/ml) in PBS was passed over the anti-GST surface of one flow cell while recombinant GST (10–30 µg/ml) was passed over the other flow cell to provide a reference surface. Increasing concentrations of rClfB_201–542_ in PBS were passed in succession over the surface of the chip without regeneration [55]–[56]. All sensorgram data were subtracted from the corresponding data from the reference flow cell. The response generated from injection of buffer over the chip was also subtracted. Data was analysed using the BIAevaluation software version 3.0. A plot of the level of binding (response units) at equilibrium against concentration of rClfB_201–542_ was used to determine the K*_D_*_._ The data shown is representative of 3 individual experiments each performed on 2 individual CM5 sensor chips.

### Bacterial adherence to human desquamated epithelial cells

Nasal desquamated epithelial cells were harvested from the anterior nares of healthy human volunteers and were prepared using a previously described protocol [19]. *S. aureus* was grown to exponential phase, harvested, washed and adjusted to 1×10^8^ cells/ml. 150 µl of bacterial cells were incubated with recombinant GST (30 µM), recombinant loricrin L2v-GST (30 µM) or an equivalent volume of PBS at room temperature for 30 minutes. 100 µl bacterial cells were then mixed with 100 µl nasal cells for 1 h at 37°C. Nasal cells were then collected, washed, stained and enumerated as previously described [19].

### Protein extraction from nasal tissue

Murine nasal tissue was excised from euthanized WT and Lor^−/−^ mice and was homogenised in 500 µl PBS. Homogenised nasal tissue was diluted 2-fold in final sample buffer (Laemmli, Sigma), and heated to 95°C for 10 min. The total protein concentration of each nose homogenate was measured using a bicinchoninic acid assay (BCA assay) and was normalised to 500 µg/ml. Samples were then analysed by Western Immunoblotting using rabbit anti-murine loricrin polyclonal IgG followed by HRP-conjugated goat anti-rabbit IgG. Bound antibody was removed by incubating at 50°C in stripping buffer (2%(w/v) sodium dodecyl sulfate, 100 mM β-mercatoethanol, 50 mM Tris-HCl, pH 6.8) and then re-probed with rabbit anti-murine K10 IgG followed by HRP-conjugated protein A. Gels were Coommassie-stained to confirm that equal protein concentrations were loaded for each sample (data not shown).

### *S. aureus* nasal colonisation models

Specific pathogen-free female FVB wildtype and FVB loricrin-deficient (Lor^−/−^) mice were housed in groups of 5 animals. Mice (8–12 weeks) were given sterile distilled water containing 500 µg/ml streptomycin or 10 µg/ml erythromycin (for *L. lactis* colonisation) 24 hours prior to nasal inoculation and for the duration of the experiment. The *S. aureus* inocula were prepared by growing cultures for 18 h on TSA, washing cells in PBS and resuspending cells in PBS containing 5% (w/v) BSA and 20% (v/v) DMSO before being frozen in small aliquots at −80°C. A single sample was thawed and cells were washed in PBS prior to inoculation. *L. lactis* MG1363 (pKS80) and *L. lactis* MG1363 (pKS80:*clfB*) were grown for 18 h in BHI containing erythromycin (10 µg/ml) and cells were washed in PBS prior to inoculation. Mice were inoculated intra-nasally with 2×10^8^ CFU of *S. aureus* or 2×10^11^ CFU *L. lactis* (10 µl per nostril).

At specific time points post inoculation mice were euthanized. The area surrounding the nose was wiped with 70% ethanol and the nose was excised and homogenised in 500 µl PBS. Lungs were also excised and homogenised in 5 ml PBS. The nose and lung homogenates were plated onto 5% horse blood agar (HBA) plates to obtain a total count of the nasal flora, and on TSA containing 500 µg/ml streptomycin or BHI containing erythromycin (10 µg/ml) to obtain the number of *S. aureus* and *L. lactis* CFU respectively, per nose and lungs.

For *in vivo* blocking studies *S. aureus* was pre-incubated with recombinant GST or recombinant L2v-GST (30 µM) for 30 mins at room temperature before intra-nasal administration. On days 1 and 2 post inoculation, mice were administered 10 µl of recombinant L2v or recombinant GST intra-nasally. Nasal tissue was then harvested on day 3 to assess bacterial burden as described above.

### Statistical analysis

Statistical analysis was performed using Prism Graphpad 5 software. Adherence and binding was analysed using an unpaired t test. Statistical analysis on nasal colonisation data was performed using a Mann-Whitney test or the Krustal-Wallis test. Pairwise comparisons for multiple groups were made using Dunns Multiple Comparisons test.

## Supporting Information

Figure S1 **Schematic representation of ClfB and sequences of minimal ClfB binding regions of keratin.** (A) Schematic representation of full length ClfB depicting locations of signal sequence (S), binding region A with subdomains (N1, N2, N3), SD-repeat region (R), wall-spanning region (W), LPETG motif, membrane anchor (M) and cytoplasmic domain (C). (B) Schematic representation of recombinant ClfB A region used in this study. The recombinant protein spans amino acids 201–542 and contains an N-terminal his-tag as indicated. Amino acid sequences of the minimal binding regions of human (C) and murine (D) K10 generated in this study.
(TIFF)

Figure S2 **Complementation of the** ***clfB*** **mutation.** *S. aureus* Newman, Newman Δ*clfB*, Newman Δ*clfB* (pCU1:*clfB*) and Newman Δ*clfB* (pCU1) were grown to exponential phase and added to wells coated with immobilized GST-HLor (1 µM). Bacterial adherence was detected by staining with crystal violet staining and measurement of the absorbance at 570 nm. Values represent the mean ± SD of triplicate wells. The data shown is representative of 2 individual experiments.
(TIFF)

Figure S3 **Recombinant loricrin.** Models of human (A) and murine (B) loricrin (adapted from [37]) depicting N- and C- terminal as well as internal regions and glycine-serine-rich loop regions. Loop regions 1A, 1B, 2, and 3 in human loricrin are highlighted in orange, green, blue and purple, respectively. Markers are included to indicate the beginning and end of each synthesized region (L1–L4). GST-tagged and purified recombinant human loricrin (C), murine loricrin (D) and loop region proteins (E) are shown on 12% SDS-PAGE gels.
(TIFF)

Figure S4 **Squamous cell adherence assay using** ***S. aureus*** **grown in iron-limited conditions.** *S. aureus* strains were grown to exponential phase in RPMI. Washed cells were incubated with recombinant GST or recombinant L2v-GST, or just resuspended in PBS, before being incubated with human nasal epithelial cells. Adherent bacteria were enumerated by microscopy and were expressed as a percentage of the positive control. Results are expressed as the mean ± SD of 3 independent experiments. Statistical analysis was performed using an unpaired t test.
(TIFF)

Figure S5 **Nasal colonisation in FVB wildtype and Lor^−/−^ mice.** Mice were inoculated intra-nasally with *S. aureus* Newman (2×10^8^ CFU). Mice were euthanized and bacterial burden in the noses established on days 3 and 10 (A). *S. aureus* Newman was pre-incubated with recombinant GST or recombinant L2v-GST for 30 min before intra-nasal inoculation (2×10^8^ CFU). Mice were then intra-nasally treated with recombinant GST or recombinant L2v-GST on days 1 and 2. Mice were euthanized and bacterial burden in the noses established on day 3 (B). Each dot indicates the number of CFU/nose for a single mouse. Results expressed as Log CFU per nose, median indicated by bar (n = 15–20 per group). Statistical analysis was performed using the Mann-Whitney test.
(TIFF)

Figure S6 **Nasal colonisation of SH1000 and SH1000 Δ** ***clfB*** **^−^ in the FVB wild-type and Lor^−/−^ mice.** Mice were inoculated intra-nasally with SH1000 or SH1000Δ*clfB* (2×10^8^ CFU). After 10 days, mice were euthanized and bacterial burden in the noses was established. Each dot indicates the number of CFU/nose for a single mouse. Results expressed as Log CFU per nose, median indicated by bar (n = 4 per group).
(TIFF)

Figure S7 **Validation of** ***S. aureus*** **Sm^r^ Newman and Newman Sm^r^ Δ** ***clfB*** **in comparison to their parental strains.** Newman was compared to Newman Δ*clfB* by performing growth curve experiments (A). Western Immunoblotting using anti-ClfB A region IgG followed by HRP-conjugated protein A was performed to compare the level of ClfB expressed by Sm^r^ Newman with the parental strain (B) and with Newman Δ*clfB* (C).
(TIFF)

Table S1 **Synthetic oligonucleotide primers used to amplify** ***clfB*** **gene fragment,** ***lor*** **Loop region 2v gene fragment,** ***clfB*** **deletion construct and** ***isdA*** **deletion construct.**
(DOC)
