# Supplementary figures and images for: Nasal Colonisation by Staphylococcus aureus Depends upon Clumping Factor B Binding to the Squamous Epithelial Cell Envelope Protein Loricrin
Source: PLoS Pathog. 2012 Dec 27;8(12):e1003092. doi: 10.1371/journal.ppat.1003092 (PMC3531522; doi:10.1371/journal.ppat.1003092)

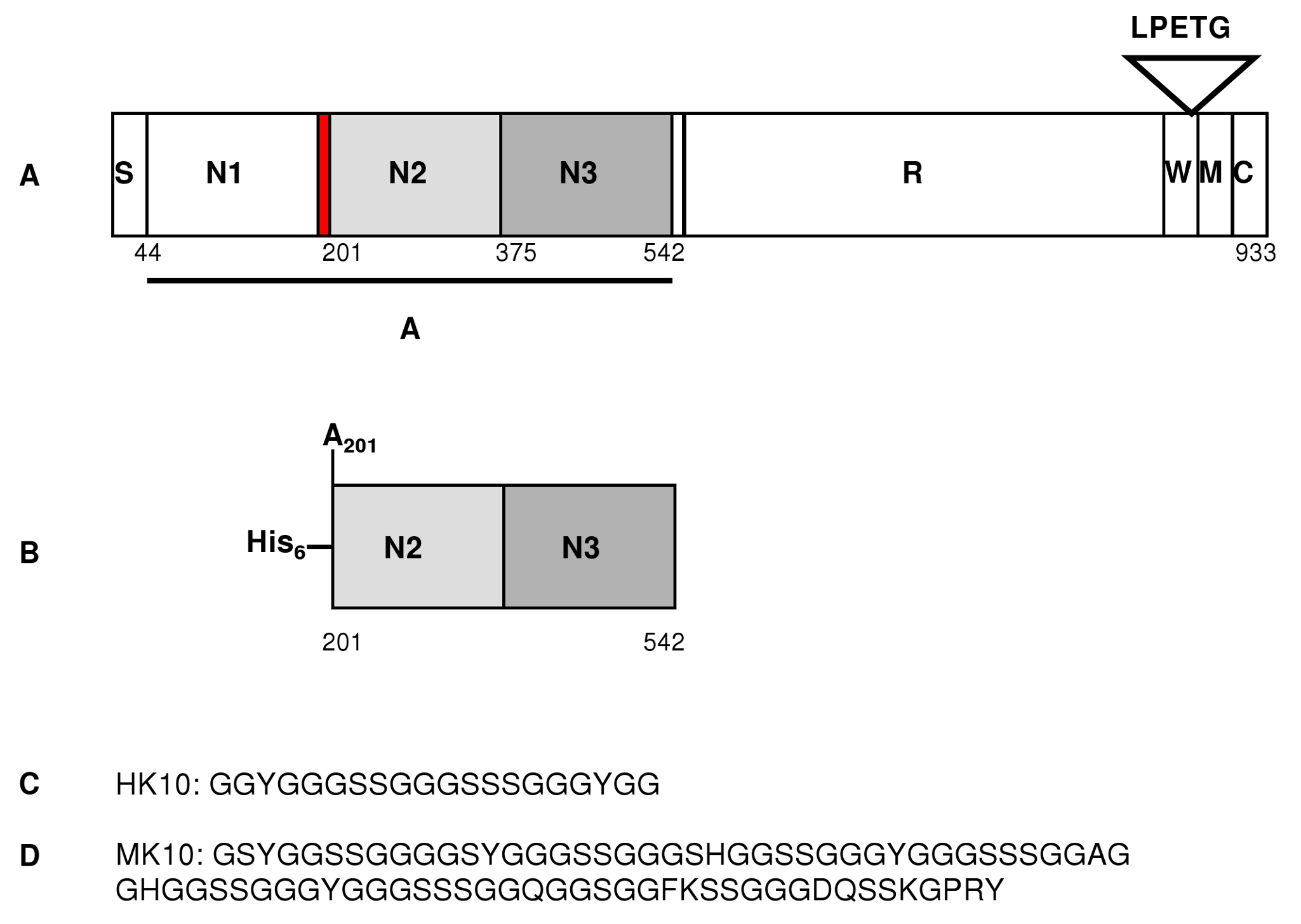

Supplement: Figure S1 — Schematic representation of ClfB and sequences of minimal ClfB binding regions of keratin. (A) Schematic representation of full length ClfB depicting locations of signal sequence (S), binding region A with subdomains (N1, N2, N3), SD-repeat region (R), wall-spanning region (W), LPETG motif, membrane anchor (M) and cytoplasmic domain (C). (B) Schematic representation of recombinant ClfB A region used in this study. The recombinant protein spans amino acids 201–542 and contains an N-terminal his-tag as indicated. Amino acid sequences of the minimal binding regions of human (C) and murine (D) K10 generated in this study. (TIFF) [file ppat.1003092.s001.tiff]

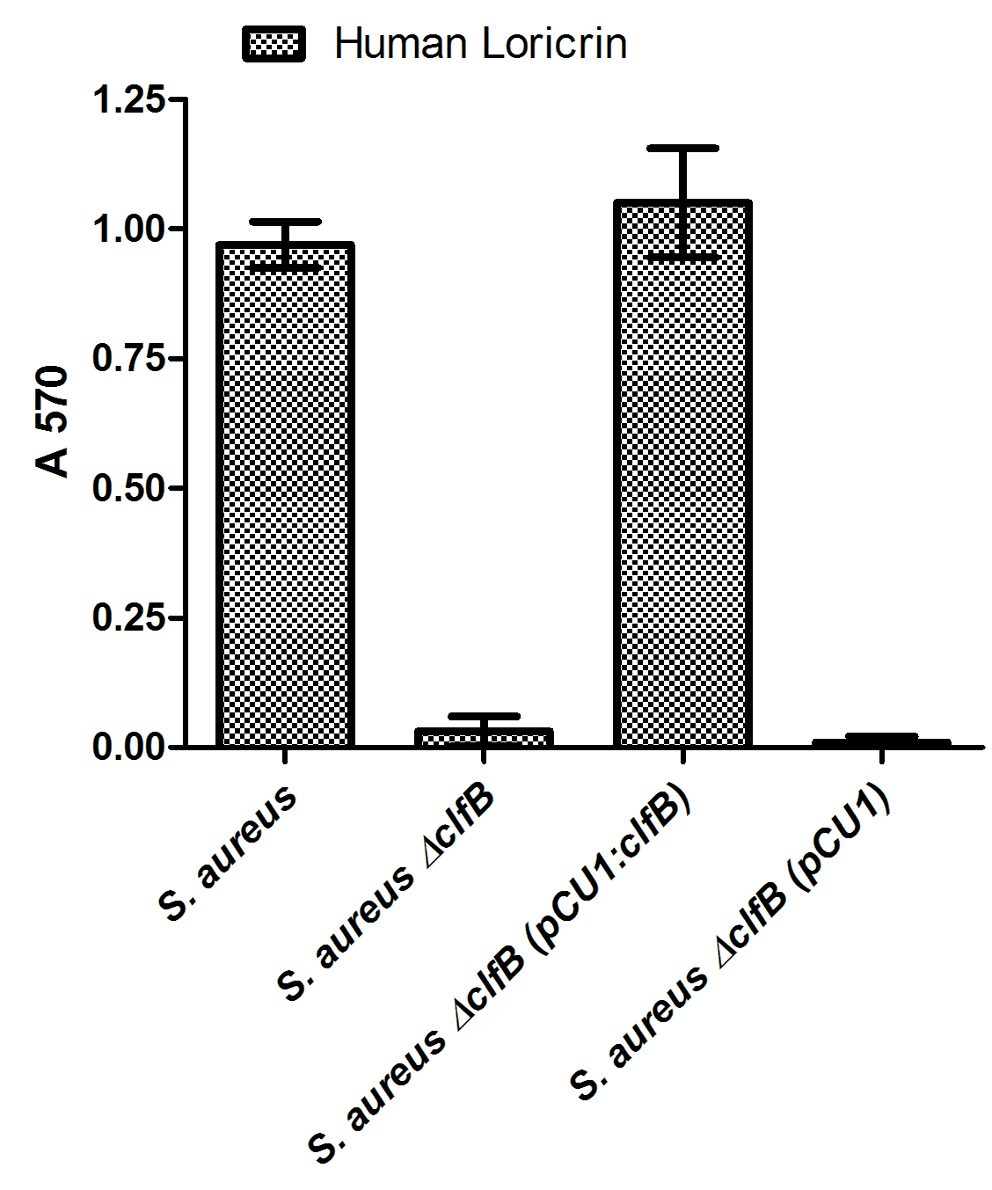

Supplement: Figure S2 — Complementation of the clfB mutation. S. aureus Newman, Newman ΔclfB, Newman ΔclfB (pCU1:clfB) and Newman ΔclfB (pCU1) were grown to exponential phase and added to wells coated with immobilized GST-HLor (1 µM). Bacterial adherence was detected by staining with crystal violet staining and measurement of the absorbance at 570 nm. Values represent the mean ± SD of triplicate wells. The data shown is representative of 2 individual experiments. (TIFF) [file ppat.1003092.s002.tiff]

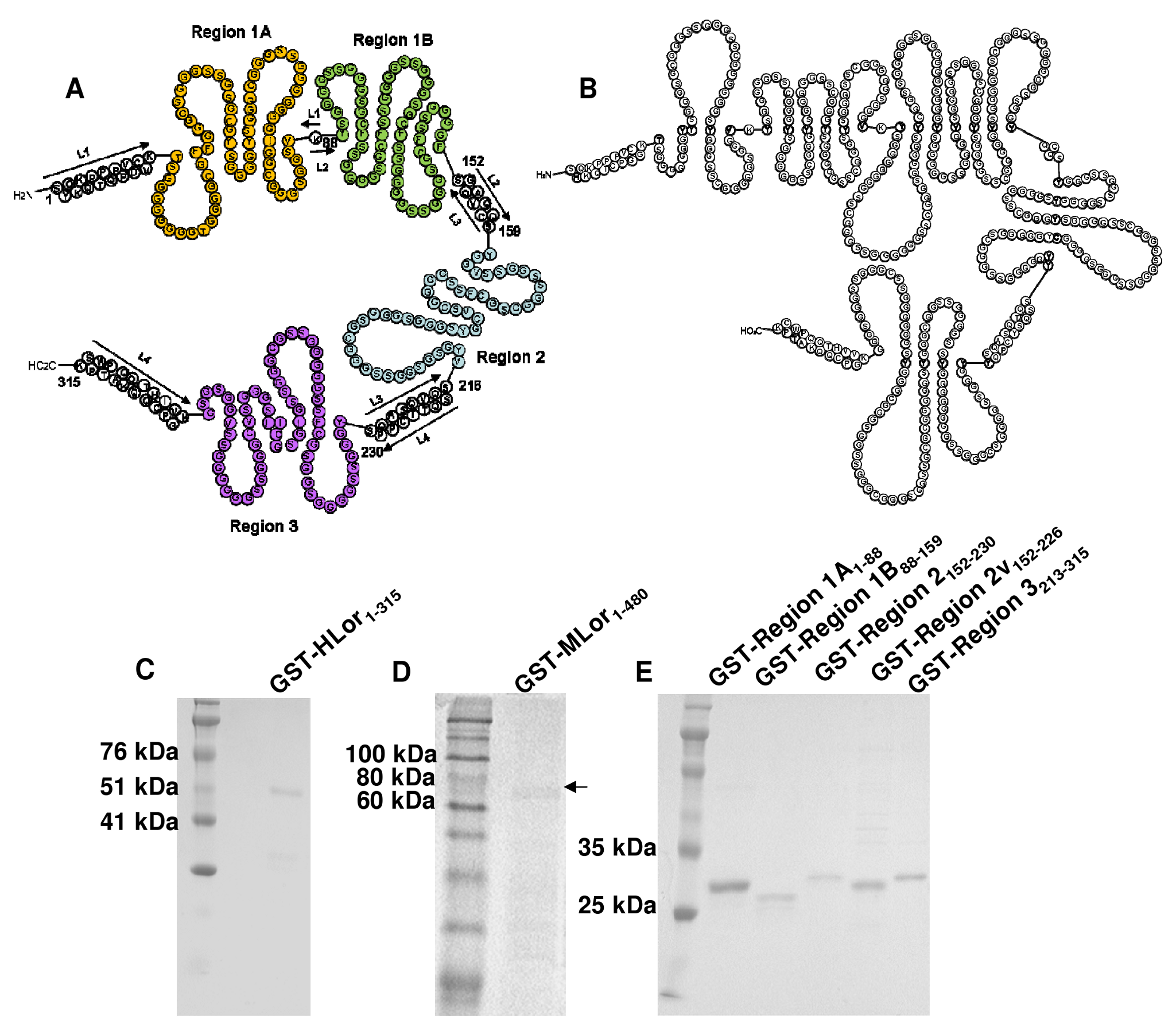

Supplement: Figure S3 — Recombinant loricrin. Models of human (A) and murine (B) loricrin (adapted from [37]) depicting N- and C- terminal as well as internal regions and glycine-serine-rich loop regions. Loop regions 1A, 1B, 2, and 3 in human loricrin are highlighted in orange, green, blue and purple, respectively. Markers are included to indicate the beginning and end of each synthesized region (L1–L4). GST-tagged and purified recombinant human loricrin (C), murine loricrin (D) and loop region proteins (E) are shown on 12% SDS-PAGE gels. (TIFF) [file ppat.1003092.s003.tiff]

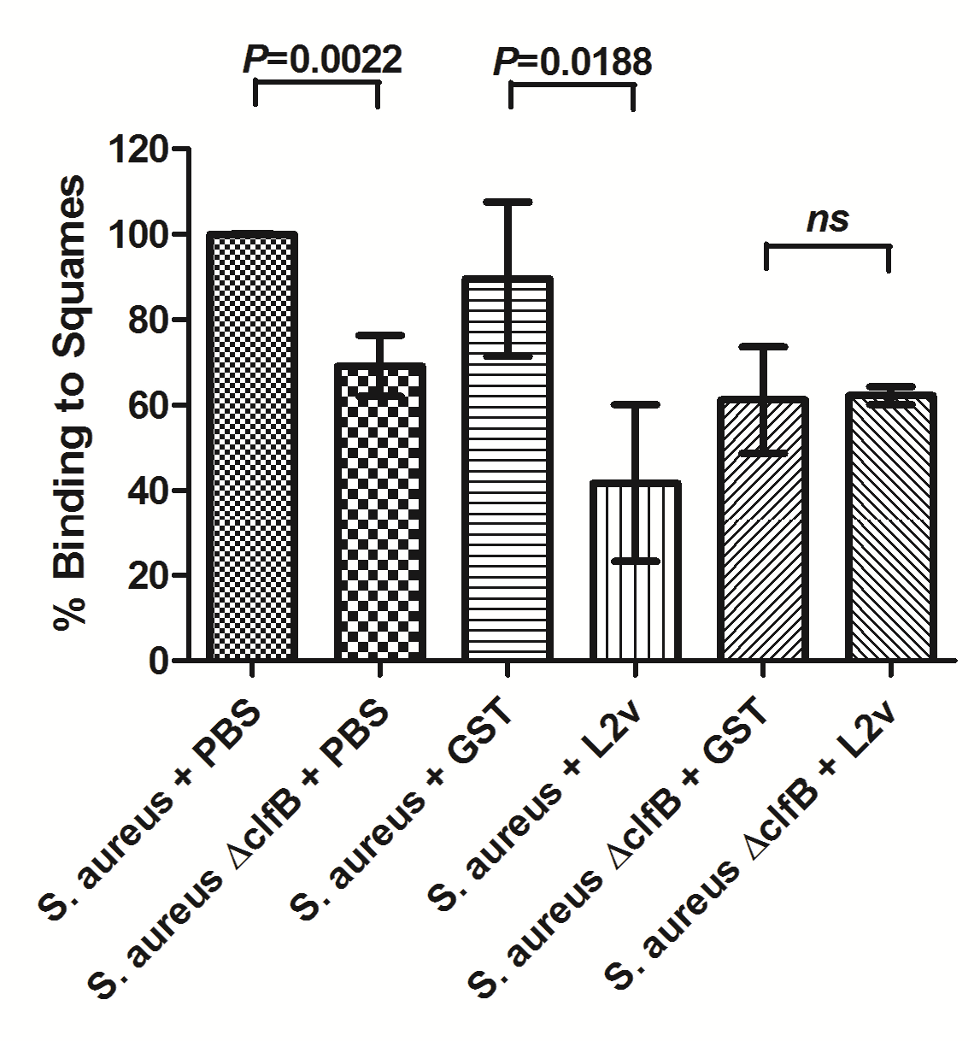

Supplement: Figure S4 — Squamous cell adherence assay using S. aureus grown in iron-limited conditions. S. aureus strains were grown to exponential phase in RPMI. Washed cells were incubated with recombinant GST or recombinant L2v-GST, or just resuspended in PBS, before being incubated with human nasal epithelial cells. Adherent bacteria were enumerated by microscopy and were expressed as a percentage of the positive control. Results are expressed as the mean ± SD of 3 independent experiments. Statistical analysis was performed using an unpaired t test. (TIFF) [file ppat.1003092.s004.tiff]

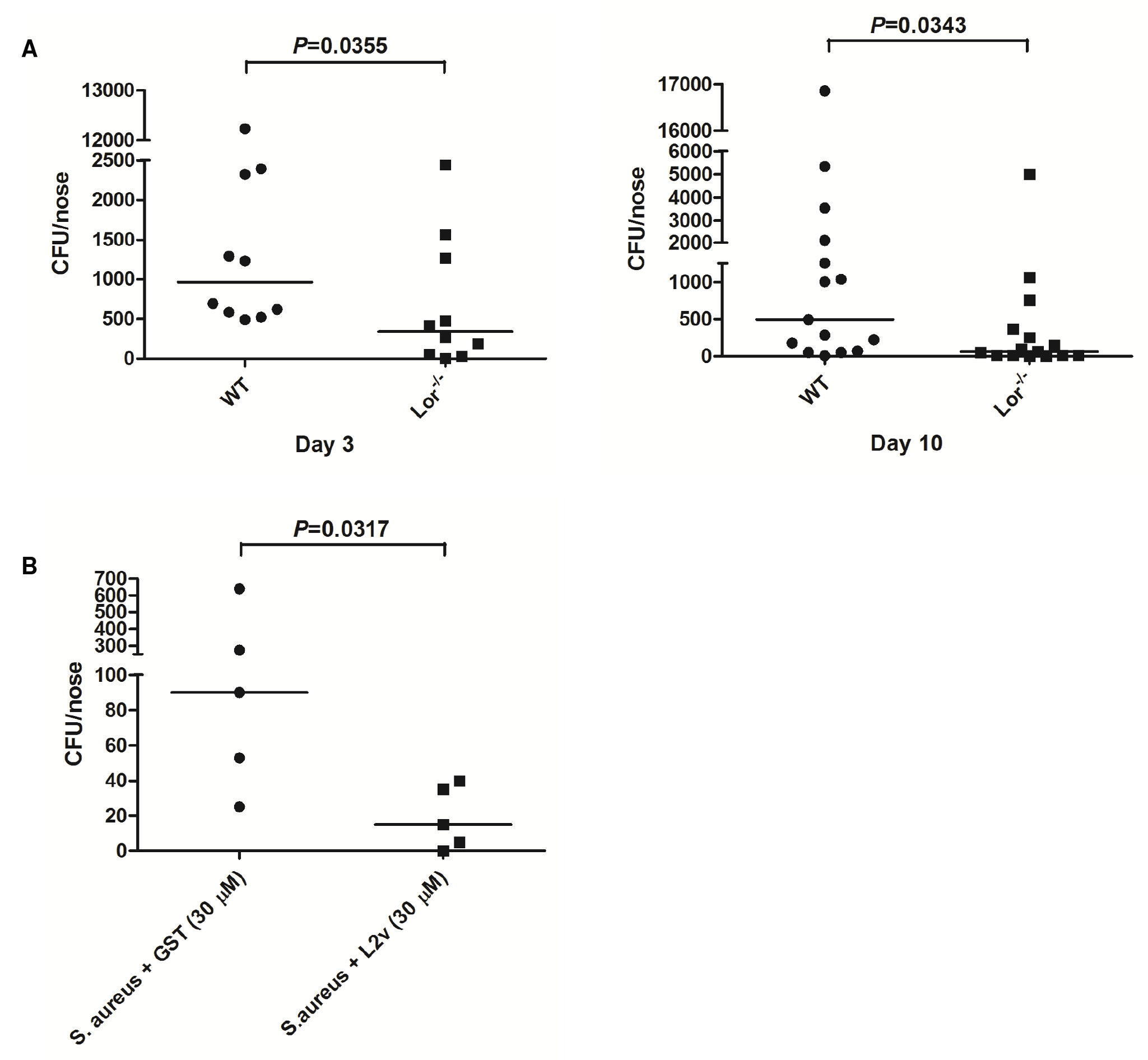

Supplement: Figure S5 — Nasal colonisation in FVB wildtype and Lor−/− mice. Mice were inoculated intra-nasally with S. aureus Newman (2×10^8^ CFU). Mice were euthanized and bacterial burden in the noses established on days 3 and 10 (A). S. aureus Newman was pre-incubated with recombinant GST or recombinant L2v-GST for 30 min before intra-nasal inoculation (2×10^8^ CFU). Mice were then intra-nasally treated with recombinant GST or recombinant L2v-GST on days 1 and 2. Mice were euthanized and bacterial burden in the noses established on day 3 (B). Each dot indicates the number of CFU/nose for a single mouse. Results expressed as Log CFU per nose, median indicated by bar (n = 15–20 per group). Statistical analysis was performed using the Mann-Whitney test. (TIFF) [file ppat.1003092.s005.tiff]

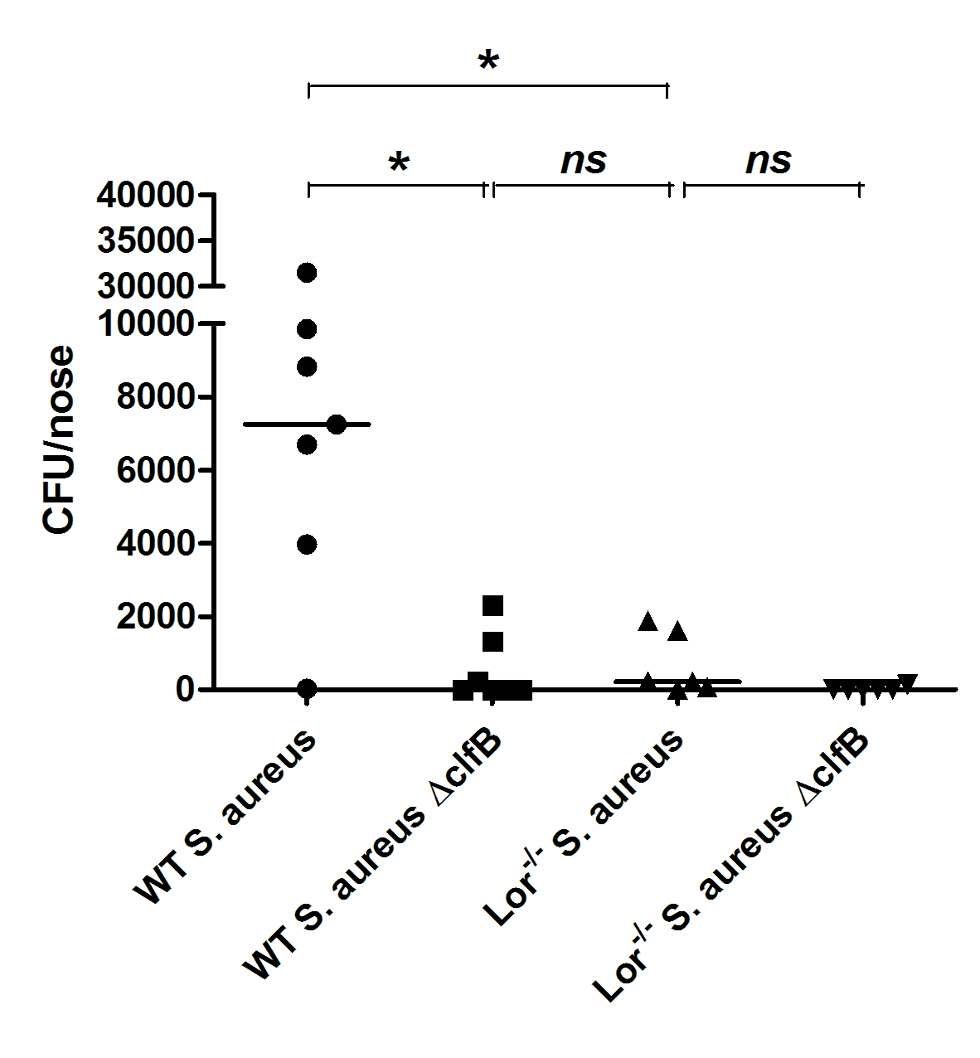

Supplement: Figure S6 — Nasal colonisation of SH1000 and SH1000 Δ clfB − in the FVB wild-type and Lor−/− mice. Mice were inoculated intra-nasally with SH1000 or SH1000ΔclfB (2×10^8^ CFU). After 10 days, mice were euthanized and bacterial burden in the noses was established. Each dot indicates the number of CFU/nose for a single mouse. Results expressed as Log CFU per nose, median indicated by bar (n = 4 per group). (TIFF) [file ppat.1003092.s006.tiff]

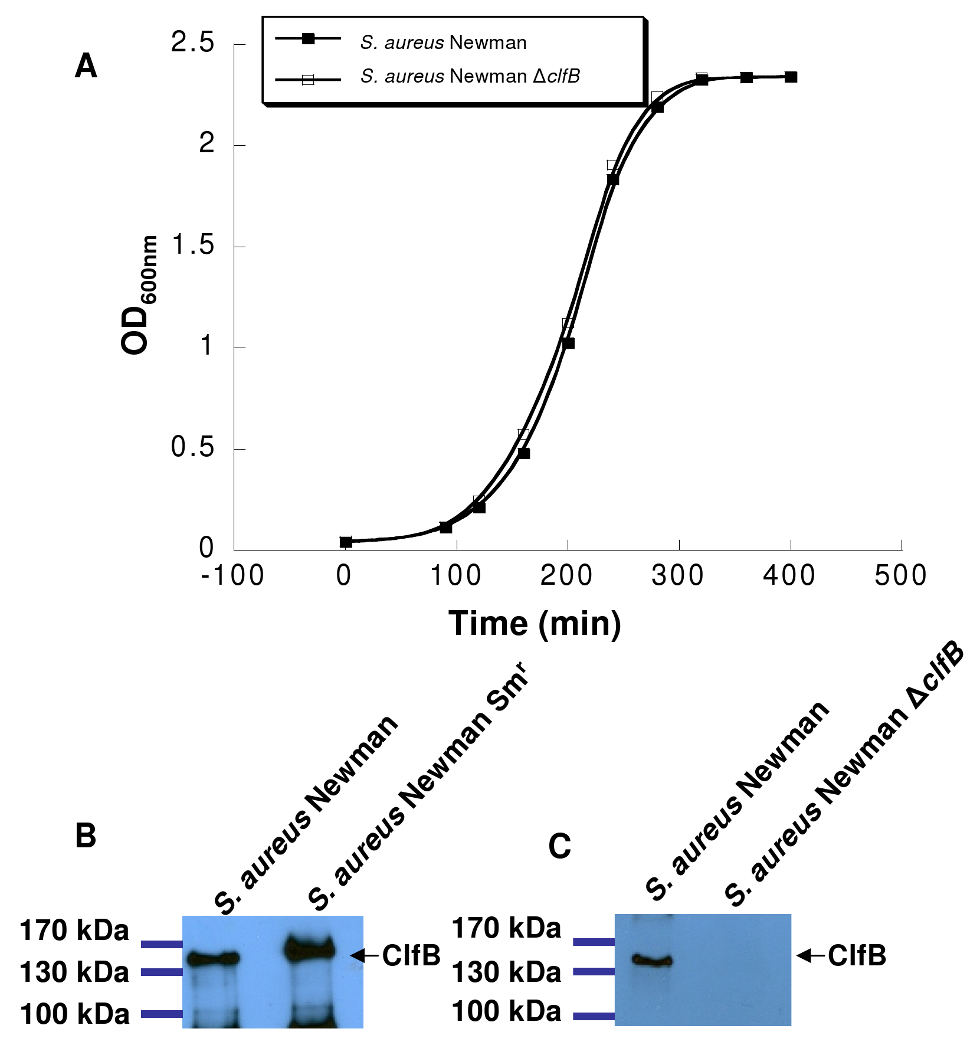

Supplement: Figure S7 — Validation of S. aureus Smr Newman and Newman Smr Δ clfB in comparison to their parental strains. Newman was compared to Newman ΔclfB by performing growth curve experiments (A). Western Immunoblotting using anti-ClfB A region IgG followed by HRP-conjugated protein A was performed to compare the level of ClfB expressed by Smr Newman with the parental strain (B) and with Newman ΔclfB (C). (TIFF) [file ppat.1003092.s007.tiff]
