# Supplementary material for: Nasal Colonisation by Staphylococcus aureus Depends upon Clumping Factor B Binding to the Squamous Epithelial Cell Envelope Protein Loricrin
Source: PLoS Pathog. 2012 Dec 27;8(12):e1003092. doi: 10.1371/journal.ppat.1003092 (PMC3531522; doi:10.1371/journal.ppat.1003092)
Supplement: Table S1 — Synthetic oligonucleotide primers used to amplify clfB gene fragment, lor Loop region 2v gene fragment, clfB deletion construct and isdA deletion construct. (DOC) [file ppat.1003092.s008.doc]

**Table S1:**

| **Constructs** | **Amino**  **Acids** | **Sequence^#^** |
| --- | --- | --- |
| ClfB N2N3 | 201-542 | 5’-GGGGGATCCGCTGAACCGGTAGTAAATG-3’ |
|  |  | 5’-GGGAAGCTTATTTACTGCTGAATCACCATC-3’ |
| Δ *clf*B cassette (A) |  | 5’-CCCGTCGACACAGTTTTTAACTATTCAACTCATGAG-3’ |
| Δ *clf*B cassette (B) |  | 5’-CAAAAATATTACTCCATTTCAATTTCTAGA-3’ |
| Δ *clf*B cassette (C) |  | 5’-AATTGAAATGGAGTAATATTTTTGTAAATACTTTTTTAGGCCGAATAC-3’ |
| Δ *clf*B cassette (D) |  | 5’-CCCGAATTCCCATATCCTCCCATAGAGTGACCT-3’ |
| Δ *isd*A cassette (A) |  | 5’-CCCGTCGACGTTGGCAGTGTTTTTACTAATAATATTTTC-3’ |
| Δ *isd*A cassette (B) |  | 5’-GTTGTTTTCCTCCTAAGGATACAA-3’ |
| Δ *isd*A cassette (C) |  | 5’-TATCCTTAGGAGGAAAACAACATCATCGTCACACTCATAACT-3’ |
| Δ *isd*A cassette (D) |  | 5’-CCCGAATTCTTCTTTTGTTTCAGAAGTAGGGGCCTC-3’ |
| Loop Region 2v | 152-230 | 5’-GGGGGATCCTCCTCGGGCCAGGCGGTCCAG-3’ |
|  |  | 5’-GGGGAATTCTTAGACCTGCTGCGAGGAGAC-3’ |

^#^ Restriction endonuclease sites are underlined
